# Proteomics of Patient-Derived Striatal Medium Spiny Neurons in Multiple System Atrophy

**DOI:** 10.3390/cells14171394

**Published:** 2025-09-06

**Authors:** Nadine J. Smandzich, Andreas Pich, Thomas Gschwendtberger, Stephan Greten, Lan Ye, Martin Klietz, Alessio Di Fonzo, Lisa M. Henkel, Florian Wegner

**Affiliations:** 1 Department of Neurology, Hannover Medical School, 30625 Hannover, Germany; 2 Center for Systems Neuroscience Hannover (ZSN), 30625 Hannover, Germany; 3 Research Core Facility Proteomics, Institute of Toxicology, Hannover Medical School, 30625 Hannover, Germany; 4 Neurology Unit, IRCCS Foundation Ca’ Granda Ospedale Maggiore Policlinico, 20122 Milan, Italy

**Keywords:** multiple system atrophy, proteomics, iPSC, striatal GABAergic medium spiny neurons, proteome analysis

## Abstract

The rare and rapidly progressive neurodegenerative disease multiple system atrophy (MSA) mainly affects the striatum and other subcortical brain regions. In this atypical Parkinsonian syndrome, the protein alpha-synuclein aggregates and misfolds in neurons as well as glial cells and is released in elevated amounts by hypoexcitable neurons. Mitochondrial dysregulation affects the biosynthesis of coenzyme Q10 and the activity of the respiratory chain, as shown in an induced pluripotent stem cell (iPSC) model. Proteome studies of cerebrospinal fluid and brain tissue from MSA patients yielded inconsistent results regarding possible protein changes due to small and combined groups of atypical Parkinsonian syndromes. In this study, we analysed the cellular proteome of MSA patient-derived striatal GABAergic medium spiny neurons. We observed 25 significantly upregulated and 16 significantly downregulated proteins in MSA cell lines compared to matched healthy controls. Various protein types involved in diverse molecular functions and cellular processes emphasise the multifaceted pathomechanisms of MSA. These data could contribute to the development of novel disease-modifying treatment strategies for MSA patients.

## 1. Introduction

In the rare neurological disease multiple system atrophy (MSA), mainly subcortical areas of the brain can be affected by cell degeneration. A more pronounced degeneration in the striatum, Parkinsonian-type (MSA-P), or in the cerebellum, cerebellar type (MSA-C), can be described in this movement disorder. Accordingly, predominantly autonomic, Parkinsonian and cerebellar symptoms may occur, for which there are only limited treatment options [1,2,3,4,5].

Although not identical to Parkinson’s disease (PD), alpha-synuclein pathology also occurs in MSA, resulting in misfolding and aggregation of the chaperone protein in the cytoplasm of oligodendrocytes and neurons [2,4,6]. An increase in neuronal release and distribution of alpha-synuclein was also shown in induced pluripotent stem cell-derived hypoexcitable striatal GABAergic medium spiny neurons from MSA-P patients compared to matched healthy controls [7]. The pathophysiology also includes neuroinflammation with activation of microglial cells and inflammatory signalling pathways [1,2,4]. Genetic studies could not identify any specific mutations leading to MSA [1,2,4].

In neurodegenerative diseases, mitochondrial processes and autophagy are often disrupted [8,9,10,11]. MSA is associated with reduced activity of the mitochondrial respiratory chain and increased biosynthesis of coenzyme Q10 [12,13,14].

Proteome studies in cerebrospinal fluids (CSF) and post-mortem brains of patients with MSA, PD and other atypical Parkinsonian syndromes such as the tauopathy progressive supranuclear palsy (PSP), corticobasal syndrome (CBS) and the synucleopathy dementia with Lewy bodies (DLB) were analysed in comparison to healthy control subjects or among themselves [6,15,16,17,18,19,20,21]. In various studies, a large number of altered proteins were detected, primarily using mass spectrometry. However, the proteins reported differed from study to study. Nevertheless, the proteins amyloid-beta precursor protein (*APP*), neuronal cell adhesion molecule (*NRCAM*) and secretogranin-2 (*SCG2*) were repeatedly reduced in CSF compared to PD and healthy control subjects [16,17]. In both studies, atypical Parkinsonian syndromes were pooled, so these observations apply to the MSA, PSP and CBS groups.

Studies of the prefrontal cortex and cingulate gyrus, in which the individual disorders were not grouped as atypical Parkinsonian syndromes, also revealed protein differences [6,18,19]. A Danish [18] and a Norwegian [19] research group analysed the proteome of the prefrontal cortex and compared the results of MSA patients with those of healthy control subjects. However, no common protein alteration was found in either study, which could be explained by differences in methodological implementation and equipment. In another recently published study, the proximal proteome of alpha-synuclein in the forebrain and midbrain of MSA patients was compared with that of PD and DLB patients. The proteins NADPH-dependent carbonyl reductase 1 (*CBR1*), alpha-crystallin B chain (*CRYAB*) and glial fibrillary acidic protein (*GFAP*) were enriched in MSA [20].

Current proteome studies provide initial approaches for the discovery of biomarkers and insights into the altered proteome of the brain in MSA patients. Nevertheless, this research still faces many challenges, as misdiagnosis, the grouping of several diseases, small patient cohorts, especially in rare, atypical Parkinsonian syndromes, and different methodologies lead to difficult interpretations. In addition to biomarkers, required for the early and reliable differentiation of the neurodegenerative diseases MSA, PD, PSP and DLB [3,4,14,22], basic research is also essential to gain further pathophysiological insights for the development of novel disease-modifying therapies.

This study focuses on the proteome analysis of cell lines from MSA-P patients (*n* = 3) compared to matched healthy controls (*n* = 3). Human induced pluripotent stem cells were differentiated into striatal GABAergic medium spiny neurons, and the proteomes were analysed using liquid chromatography-mass spectrometry. A total of 151 proteins differed in their expression between the cell lines from MSA-P patients and the controls.

## 2. Materials and Methods

### 2.1. Cell Culture and Striatal GABAergic Medium Spiny Neuron (MSN) Differentiation

In this study, human induced pluripotent stem cell (hiPSC) lines from three MSA-P patients, and three matched healthy controls were used as a cell model (Table 1), which had been characterised in previous works [7,13]. Skin punch biopsies from MSA-P patients (cell lines P2 and P3) and healthy subjects (cell lines CTR1 and CTR3) were taken after written informed consent. Ethical approval for the generation of these cell lines was granted by the Ethics Committee of Hannover Medical School (No. 8666_BO_K_2019, date of approval: 13 September 2019).

The cultivation of hiPSCs was carried out in mTeSR Plus medium (STEMCELL Technologies, Vancouver, BC, Canada, 100-0276) with 1% Penicillin-Streptomycin (Thermo Fisher Scientific Inc., Waltham, MA USA, 15140122) on hESC-qualified Matrigel matrix (Corning, Now York, NY, USA, 354277). Cells were passaged as clumps using 0.5 mM EDTA (Thermo Fisher Scientific Inc., AM9260G) and mTeSR Plus with 10 µM ROCK inhibitor (Y-27632, STEMCELL Technologies, 72304).

Striatal GABAergic medium spiny neuron differentiation was performed after adaption from Henkel et al. [7] and Staege et al. [23]. Starting on day 0 as embryoid bodies (EBs), hiPSCs were passaged in mTeSR Plus with 1 µM dorsomorphin dihydrochloride (D, Abcam, Cambridge, UK, ab144821), 10 µM SB 431542 (SB, Abcam, ab291112), 1 µM Wnt Antagonist II (IWP-2, Merck KGaA, Darmstadt, Germany, 681671) and 10 µM Y-27632. The basal medium from day 2 to 11 (N2 medium) consisted of KnockOut DMEM/F-12 (Thermo Fisher Scientific Inc., 12660012), N-2 Supplement (Thermo Fisher Scientific Inc., 17502048), GlutaMAX Supplement (Thermo Fisher Scientific Inc., 35050038), 1% MEM Non-Essential Amino Acids Solution (Thermo Fisher Scientific Inc., 11140035), 1% Penicillin-Streptomycin and 15 mM HEPES, (Merck KGaA, H0887). On day 2, 1 µM D, 10 µM SB and 1 µM IWP-2 were added to the N2 medium. The same concentrations and 1 µM purmorphamine (PMA, Abcam, ab120933) were supplemented on day 4. On day 6 and 8, the same two molecules and concentrations were added (1 µM IWP-2 and 1 µM PMA). No additives were required on day 10; only the N2 medium was used.

As maturation medium, DMEM/F-12 with GlutaMAX Supplement (Thermo Fisher Scientific Inc., 31331093), Neurobasal medium (Thermo Fisher Scientific Inc., 21103049), N-2 Supplement, B-27 Supplement without Vitamin A (Thermo Fisher Scientific Inc., 12587010), 1% Penicillin-Streptomycin and 1% GlutaMAX Supplement were mixed. 20 ng/mL brain-derived neurotrophic factor (BDNF, Thermo Fisher Scientific Inc., 450-02), 10 ng/mL glial cell-derived neurotrophic factor (GDNF, Thermo Fisher Scientific Inc., 450-10) and 0.05 mM dibutyryl cyclic-AMP (dbcAMP, Merck KGaA, D0260) were added fresh due to their short-term stability.

EBs were seeded in maturation medium on hESC-qualified Matrigel matrix-coated plates from 12 to 16. The cells were passaged with Accutase (Thermo Fisher Scientific Inc., A1110501) and in maturation medium containing 10 µM Y-27632 on poly-DL-ornithine hydrobromide (80 µg/mL, PORN, Merck KGaA, P8638) and laminin-coated (10 µg/mL, Thermo Fisher Scientific Inc., 23017015) dishes on days 16 to 18 and again on PORN and laminin-coated well plates on days 24 to 30. From then on, the cells matured for up to 70 ± 7 days.

### 2.2. Immunocytochemistry (ICC)

The differentiated cells were fixed on day 70 (±7) with 4% paraformaldehyde (PFA, Merck KGaA, 8.18715). After fixation for 20 min at room temperature, the cells were washed three times with phosphate-buffered saline (PBS, Thermo Fisher Scientific Inc., 14190169) and treated with a blocking solution for 60 min (1% bovine serum albumin, BSA, Merck KGaA, A7906; 5% goat serum, Thermo Fisher Scientific Inc., 16210072; 0.3% Triton X-100, Merck KGaA, T8787; in PBS). The primary antibodies prepared in the blocking solution mouse anti-beta 3 tubulin (TUJ1, 1:1000; Abcam, ab78078, RRID:AB_2256751), rabbit anti-gamma-aminobutyric acid (GABA, 1:1000; Merck KGaA, A2052, RRID:AB_477652) and rat anti-COUP TF-1 interacting protein 2 (CTIP2, 1:300; Abcam, ab18465, RRID:AB_2064130) were incubated overnight at 4 °C. Afterwards, the cells were washed again before incubating the secondary antibodies for 2 h at room temperature. Goat anti-mouse Alexa Fluor 488 (1:1000; A-21131, RRID:AB_2535771), goat anti-rabbit Alexa Fluor 555 (1:1000; A-21428, RRID:AB_2535849), goat anti-rabbit Alexa Fluor 488 (1:500; A-11034, RRID:AB_2576217) and goat anti-rat (1:500; A-21434, RRID:AB_2535855) (all Thermo Fisher Scientific Inc.) were prepared in PBS. Mowiol 4-88 (Merck KGaA, 475904) with 4′,6-diamidino-2-phenylindole (DAPI, 10 µg/mL, Thermo Fisher Scientific Inc., D1306) was applied as the embedding medium.

For the fluorescence imaging, the Olympus BX61 fluorescence microscope, the Olympus BX-UCB control box, the Olympus DP72 camera (Olympus, Hamburg, Germany), the X-Cite 120Q fluorescence lamp (Micrasys, Herborn, Germany) and the Olympus cellSens Dimension 1.18 analysis software were used. Positively stained cells were counted in four randomly selected visual areas. For each cell line and differentiation, both DAPI, TUJ1 and GABA and DAPI, GABA and CTIP2 stainings were counted separately and calculated as a percentage ratio to DAPI, TUJ1 or GABA. The percentages were summarised for the control group of three healthy control cell lines and the MSA group of three MSA-P patient cell lines.

### 2.3. Reverse Transcription Quantitative Real-Time Polymerase Chain Reaction (RT-qPCR)

The RNAs of hiPSCs and MSNs (70 ± 7 days) were isolated with RNeasy Mini Kit (QIAGEN, Venlo, The Netherlands, 74104) and transcribed into cDNA via QuantiTect Reverse Transcription Kit (QIAGEN, 205311). As recommended, the RNAs were purified with a RNase-free DNAse Set (QIAGEN, 79254). All kits were performed according to the manufacturer’s protocol. For the determination of the RNA concentrations, Nanodrop 2000c (Thermo Fisher Scientific Inc.) was used. In a StepOnePlus cycler (Applied Biosystems StepOnePlus Real-Time PCR System, StepOne Software v2.3, Thermo Fisher Scientific Inc.), real-time PCR was performed as triplicates with SYBR Green PCR Master Mix (Thermo Fisher Scientific Inc., 4367659), 7 ng cDNA, 1.75 µM forward and reverse primers (see Table S1 for the primer sequences) [7,23]. Neuronal (TUJ1; MAP2, microtubule-associated protein 2; FOXP1, forkhead box protein P1), GABAergic (GAD67, glutamic acid decarboxylase; FOXG1, forkhead box protein G1), MSN-specific (CTIP2) and three reference genes (beta-2 microglobulin, B2M; glyceraldehyde-3-phosphate dehydrogenase, GAPDH; and beta-actin, ACTB) were examined in this study. The PCR reaction was performed as follows: Start at 95 °C for 10 min, 40 cycles of 95 °C for 15 s and 60 °C for 1 min.

The gene expressions were analysed for each cell line, comparing MSNs with their corresponding hiPSCs. ΔCt Reference was calculated as the average of the Ct values of the three reference genes. ΔCt Reference was subtracted from the Ct values of the mean target genes to determine ΔCt Sample. As ΔCt Calibrator, the averaged Ct values of the hiPSCs were calculated for each cell line. The mean ΔCt Calibrator was subtracted from ΔCt Sample to calculate ΔΔCt and, accordingly, the fold expressions (2^−ΔΔCt^).

### 2.4. Liquid Chromatography-Mass Spectrometry (LC-MS) Analysis

On day 70 (±7), the cells were cooled on ice and washed at least once with pre-cooled PBS. Afterwards, MSNs were treated with 80% methanol solution (MtOH), scraped, vortexed and stored at −80 °C. After thawing, the samples were dried and dissolved in lysis buffer (8 M urea and 50 mM ammonium bicarbonate, pH 8.0).

MS-based proteome analyses were performed as described recently [24]. Briefly, proteins were mixed and alkylated by iodoacetamide, digested with trypsin, and further seperated into six fractions by alkaline reversed-phase (RP) chromatography as described. Peptide samples were analysed with data-dependent acquisition in an LC-MS system (RSLC, Orbitrap Exploris 240, both Thermo Fisher Scientific). Raw MS data were processed using MaxQuant (version 2.0, [25]) and Perseus software (version 2.0.11.0, [26]). Human entries from Universal Protein Knowledgebase (UniProtKB) were used for identification, and proteins were stated as identified with a false discovery rate (FDR) < 0.01 at the protein and peptide level. For each sample, the intensities were normalised by subtracting the corresponding median. Only proteins whose intensity was determined in at least all three biological replicates (independent differentiations) of one cell line were considered, and missing values were imputed by using normal distribution (downshift 1.8, width 0.3).

### 2.5. Proteomics

The proteome of three matched healthy control cell lines and three MSA-P patient cell lines, each with three independent differentiations, was analysed. For each cell line and protein, the log_2_ intensities were averaged. The averaged log_2_ intensities were assigned to the CTR or MSA group and statistically tested using an unpaired two-tailed *t*-test. A Benjamini–Hochberg correction was not applied. The fold change (FC) or the difference between the two groups was calculated from the total averaged log_2_ intensities of the CTR group subtracted from the total averaged log_2_ intensities of the MSA group. Data with −log_10_ *p*-values (*p*) and log_2_ FC are shown in the volcano plot. In addition, the averaged log_2_ intensities of CTR and MSA groups were visualised as heat maps for the proteins with significant downregulation (−log_10_ *p* > 1.3 and log_2_ FC < −1) and significant upregulation (−log_10_ *p* > 1.3 and log_2_ FC > 1). For seven proteins, a scatter plot was selected to visualise the averaged log_2_ intensities of the cell lines as data points. The proteome data were analysed using various bioinformatics tools.

#### 2.5.1. PANTHER Classification System

Using the PANTHER classification system (version 19.0, 26 June 2025, [27]), a statistical over-representation analysis was performed using Fisher’s exact test and Benjamini–Hochberg false discovery rate (FDR), with the whole genome of Homo sapiens as reference for all genes that were significant according to the unpaired two-tailed *t*-test (*p* < 0.05 or −log_10_ *p* > 1.3). Considering the significance criteria *p* < 0.05 and FDR < 0.05, the analysis revealed entries in biological processes, cellular components and molecular functions according to the Gene Ontology (GO) classification and the PANTHER GO-Slim classification.

For the significantly downregulated (*p* < 0.05 and log_2_ FC < −1) and upregulated proteins (*p* < 0.05 and log_2_ FC > 1), no statistically over-represented results could be obtained with the over-representation test. A functional classification was carried out for these corresponding genes, which were also classified according to GO, PANTHER pathways and protein classes.

#### 2.5.2. Reactome Database

For the reaction analysis, an over-representation test with hypergeometric distribution and FDR according to Benjamini–Hochberg was performed using the Reactome database (version 93, 1 July 2025, [28]). For the proteins that were significant in the unpaired two-tailed *t*-test, Reactome pathways were determined that were statistically significant with *p* < 0.05 and FDR < 0.05. An over-representation test with the significantly downregulated (*p* < 0.05 and log_2_ FC < −1) and upregulated proteins (*p* < 0.05 and log_2_ FC > 1) revealed no statistically over-represented Reactome pathways.

#### 2.5.3. Ingenuity Pathway Analysis (IPA)

Using Ingenuity Pathway Analysis (IPA, QIAGEN, 14 July 2025, [29]) to identify potential upstream regulators, the data for the normalised proteins, including *p*-values (unpaired two-tailed *t*-test) and log_2_ fold changes (FC), were uploaded. Of these, 23 genes were unmapped and were excluded. The Core Analysis, which provided results for the upstream regulator, the causal and mechanistic network, and the downstream effect, was performed based on the expression analysis and the experimental log ratio (log_2_ FC). The cut-off value for the *p*-value was set at 0.05, resulting in 150 analysable molecules. For statistical analysis, overlap *p*-values were calculated using one-sided Fisher’s exact test. Activation z-scores were calculated according to Krämer et al. [29], which predict the directionality of genes as activated or inhibited. For the statistical significance, results with *p* < 0.05 and z-score < −2 were considered as predicted inhibited and with *p* < 0.05 and z-score > 2 as predicted activated [29]. Only genes, RNAs and proteins were defined as molecule types for upstream molecules.

The Core Analysis was repeated with additional cut-offs for FC (−1 and 1), resulting in 40 analysable molecules. A statistical significance could not be achieved for these.

### 2.6. Statistical Analysis

Unless otherwise stated, statistical analyses were performed with GraphPad Prism 10.4.2. Cell lines were grouped according to CTR or MSA group and tested statistically using unpaired two-tailed *t*-tests. *p*-values (*p*) with *p* < 0.05 were defined as statistically significant. Graphs and scatter plots show mean values and standard error of the mean (SEM). Further statistical analyses used in the bioinformatic tools can be found in the corresponding methodology or in the figure captions.

## 3. Results

### 3.1. Striatal GABAergic Medium Spiny Neuron (MSN) Differentiation

**Figure 1:**
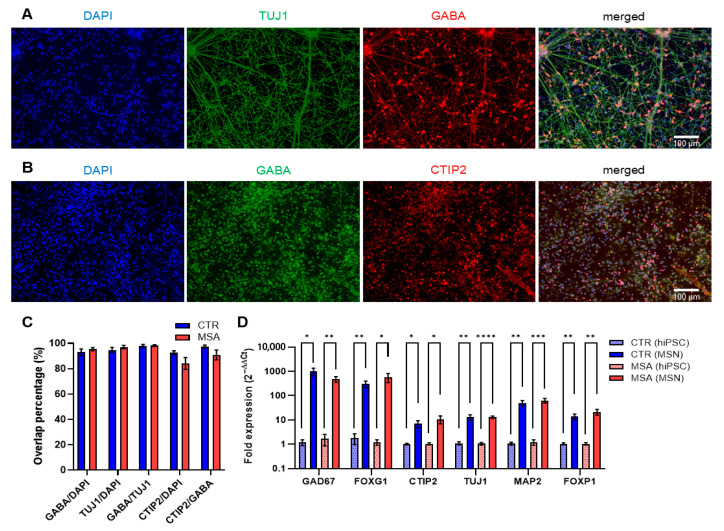
Maturation of hiPSC-derived striatal GABAergic medium spiny neurons (MSN). Representative images of MSN differentiation after 70 (±7) days (the control cell line CTR1 is displayed) show ICC staining of 4′,6-diamidino-2-phenylindole (DAPI) (blue), neuron-specific beta 3 tubulin (TUJ1) (green) and gamma-aminobutyric acid (GABA) (red) (**A**), DAPI (blue), GABA (green) and striatal COUP TF-1 interacting protein 2 (CTIP2) (red) (**B**). Scale bar indicates 100 µm (**A**,**B**). Cells from three independent differentiations of three control (CTR) and three MSA-P cell lines (MSA) were stained and counted. The counts of the positively stained cells were normalised to DAPI, TUJ1 or GABA. The mean percentages of each differentiation were summarised for the MSA (*n* = 3) and CTR (*n* = 3) groups (**C**). In the comparative gene expression analysis, the GABAergic marker gene glutamic acid decarboxylase (GAD67), forkhead box protein G1 (FOXG1), the striatal gene CTIP2 and the neuronal genes TUJ1, microtubule-associated protein 2 (MAP2) as well as forkhead box protein P1 (FOXP1) were investigated by quantitative real-time PCR (**D**). Gene expressions were compared between MSNs and their corresponding hiPSCs. The fold expressions (2^−ΔΔCt^) of two to three independent differentiations per cell line, summarised for CTR (*n* = 3) and MSA (*n* = 3), are shown. Unpaired two-tailed *t*-tests were applied (* *p* < 0.05, ** *p* < 0.01, *** *p* < 0.001, **** *p* < 0.0001) (**D**). Graphs represent means ± SEM (**C**,**D**).

To verify the 70-day differentiation of human induced pluripotent stem cells (hiPSCs) into striatal GABAergic medium spiny neurons (MSNs), the neuronal, GABAergic and striatal marker expressions were analysed in the differentiated cells of three control and three MSA-P cell lines. For this, three independent differentiations of each cell line were evaluated by immunocytochemistry (ICC) using 4′,6-diamidino-2-phenylindole (DAPI) as well as antibodies against neuron-specific beta 3 tubulin (TUJ1), gamma-aminobutyric acid (GABA) and striatal COUP TF-1 interacting protein 2 (CTIP2) (Figure 1A–C). Furthermore, we performed comparative gene expression analysis of TUJ1, microtubule-associated protein 2 (MAP2), forkhead box protein P1 (FOXP1), glutamic acid decarboxylase (GAD67), forkhead box protein G1 (FOXG1), and striatal marker gene CTIP2 (Figure 1D).

Based on the DAPI-stained cells, over 93% of the differentiated cultures expressed TUJ1 and GABA. Approximately 95% of CTR and 97% of MSA cells were TUJ1-positive, 93% of CTR and 95% of MSA cells were GABA-positive. In both groups, 98% of the TUJ1-stained neurons were also GABAergic. Comparing the staining of the striatal marker CTIP2/DAPI, fewer stained cells were observed in the MSA (84%) than in the CTR group (93%), although the difference was not significant (unpaired two-tailed *t*-test, *p* = 0.1061). Of the GABAergic-positive cells, 98% of the CTR and 91% of the MSA group expressed CTIP2 (Figure 1C).

The fold expressions (2^−ΔΔCt^) of neuronal (TUJ1, MAP2), FOXP1, GABAergic (GAD67), FOXG1 and striatal (CTIP2) genes were significantly more expressed in the differentiated MSNs than in the corresponding hiPSCs (Figure 1D).

These results confirm a homogenous cell culture of mainly hiPSC-derived striatal GABAergic medium spiny neurons from which the proteomes were investigated.

### 3.2. Proteome Analysis

**Figure 2:**
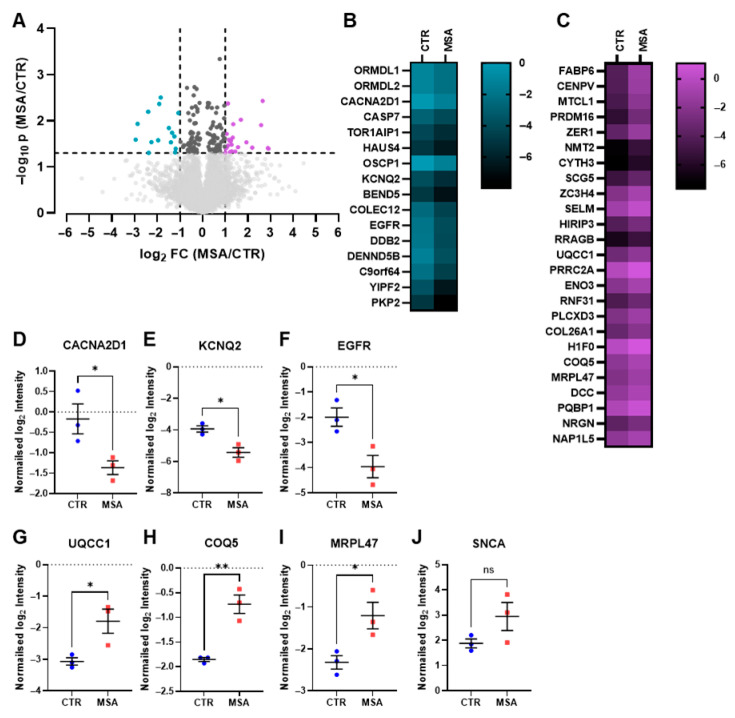
Protein expression of GABAergic medium spiny neurons from MSA-P patient and control cell lines. 5817 normalised proteins were quantified in three independent differentiations of three MSA-P patient (MSA, *n* = 3) and three control cell lines (CTR, *n* = 3) and plotted as dots in a volcano plot with −log_10_ *p*-values and log_2_ fold changes (**A**). Comparing protein expression between MSA and CTR, 151 proteins were classified as significantly different in their protein expression with *p*-values (*p*) < 0.05 (−log_10_ *p* > 1.3). The fold change (FC) indicates the direction in which the protein or gene expression is shifted. 25 proteins were upregulated in MSA cell lines, with *p* < 0.05 and log_2_ FC > 1 (at least twofold increase), shown as purple dots. 16 proteins were downregulated in MSA cell lines, with *p* < 0.05 and log_2_ FC < −1 (at least 0.5-fold decrease), shown as turquoise dots. Dark grey dots represent the remaining significant proteins (*p* < 0.05). All other protein expressions were displayed as light grey dots. Heat maps visualise the normalised mean log_2_ intensities of the downregulated (**B**) and upregulated proteins (**C**) in CTR and MSA cell lines. Darker colours indicate lower protein intensities. As examples, the three downregulated proteins *CACNA2D1* (**D**), *KCNQ2* (**E**), *EGFR* (**F**), three upregulated proteins *UQCC1* (**G**), *COQ5* (**H**) and *MRPL47* (**I**) and alpha-synuclein (*SNCA*) (*p* = 0.1415) (**J**) with their normalised log_2_ intensities are shown as a scatter plot. Each data point corresponds to the averaged and normalised log_2_ intensity of one cell line. Graphs represent means ± SEM. Unpaired two-tailed *t*-tests were applied (* *p* < 0.05, ** *p* < 0.01, ns = not significant) (**A**,**D**–**J**). *ORMDL1*: Orosomucoid 1 (ORM1)-like protein 1; *ORMDL2*: ORM1-like protein 2; *CACNA2D1*: Voltage-dependent calcium channel subunit alpha-2/delta-1; *CASP7*: Caspase-7; *TOR1AIP1*: Torsin-1A-interacting protein 1; *HAUS4*: HAUS (homologous to augmin subunit) augmin-like complex subunit 4; *OSCP1*: Organic solute carrier partner 1; *KCNQ2*: Voltage-gated potassium channel subunit Kv7.2; *BEND5*: BEN (BANP, E5R and Nac1) domain-containing protein 5; *COLEC12*: Collectin-12; *EGFR*: Epidermal growth factor receptor; *DDB2*: DNA damage-binding protein 2; *DENND5B*: DENN (differentially expressed in normal and neoplastic cells) domain-containing protein 5B; *C9orf64*: Queuosine 5′-phosphate *N*-glycosylase/hydrolase; *YIPF2*: Yip1 domain family member; *PKP2*: Plakophilin-2; *FABP6*: Gastrotropin; *CENPV*: Centromere protein V; *MTCL1*: Microtubule cross-linking factor 1; *PRDM16*: Histone-lysine *N*-methyltransferase PR-domain containing 16; *ZER1*: Protein zer-1 homologue; *NMT2*: Glycylpeptide *N*-tetradecanoyltransferase 2; *CYTH3*: Cytohesin-3; *SCG5*: Neuroendocrine protein 7B2; *ZC3H4*: Zinc finger C-x8-C-x5-C-x3-H (CCCH) domain-containing protein 4; *SELM*: Selenoprotein M; *HIRIP3*: Histone cell cycle regulator (HIRA)-interacting protein 3; *RRAGB*: Ras-related GTP-binding protein B; *UQCC1*: Ubiquinol-cytochrome-c reductase complex assembly factor 1; *PRRC2A*: Proline-rich coiled coil 2A; *ENO3*: Beta-enolase; *RNF31*: E3 ubiquitin-protein ligase RNF31; *PLCXD3*: Phosphatidylinositol-specific phospholipase C X domain containing 3; *COL26A1*: Collagen alpha-1(XXVI) chain; *H1F0*: Histone H1.0; *COQ5*: 2-methoxy-6-polyprenyl-1.4-benzoquinol methylase (mitochondrial); *MRPL47*: Large ribosomal subunit protein uL29m; *DCC*: Netrin receptor DCC (Deleted in Colorectal Carcinoma); *PQBP1*: Polyglutamine-binding protein 1; *NRGN*: Neurogranin; *NAP1L5*: Nucleosome assembly protein 1-like 5.

For the proteome analysis, three independent differentiations of three control (CTR) and three MSA-P patient cell lines (MSA) were analysed, in which a total of 6959 proteins were identified. Only proteins that were quantified in at least three biological replicates (independent differentiations) of one cell line were considered, resulting in a reduction in the number of proteins to 5817. After *t*-testing, the −log_10_ *p*-values and the log_2_ fold changes (FC) of these proteins were plotted as volcano plot (Figure 2A).

Comparing the protein expressions of MSA-P cell lines (MSA) with those of controls (CTR), 151 proteins with significantly different protein intensities were identified (*p* < 0.05, unpaired two-tailed *t*-test). A list of these proteins with gene names, *p*-values and log_2_ fold changes is provided in the Supplement (see Table S2). Of these, 25 proteins were significantly upregulated in MSA (*p* < 0.05 and log_2_ fold change > 1) (Figure 2A, purple dots). These proteins were gastrotropin (*FABP6*), centromere protein V (*CENPV*), microtubule cross-linking factor 1 (*MTCL1*), histone-lysine *N*-methyltransferase PR-domain containing 16 (*PRDM16*), protein zer-1 homologue (*ZER1*), glycylpeptide *N*-tetradecanoyltransferase 2 (*NMT2*), cytohesin-3 (*CYTH3*), neuroendocrine protein 7B2 (*SCG5*), zinc finger C-x8-C-x5-C-x3-H (CCCH) domain-containing protein 4 (*ZC3H4*), selenoprotein M (*SELM*), histone cell cycle regulator (HIRA)-interacting protein 3 (*HIRIP3*), Ras-related GTP-binding protein B (*RRAGB*), ubiquinol-cytochrome-c reductase complex assembly factor 1 (*UQCC1*), proline-rich coiled coil 2A (*PRRC2A*), beta-enolase (*ENO3*), E3 ubiquitin-protein ligase RNF31 (*RNF31*), phosphatidylinositol-specific phospholipase C X domain containing 3 (*PLCXD3*), collagen alpha-1(XXVI) chain (*COL26A1*), histone H1.0 (*H1F0*), 2-methoxy-6-polyprenyl-1.4-benzoquinol methylase (mitochondrial; *COQ5*), large ribosomal subunit protein uL29m (*MRPL47*), netrin receptor DCC (Deleted in Colorectal Carcinoma, *DCC*), polyglutamine-binding protein 1 (*PQBP1*), neurogranin (*NRGN*) and nucleosome assembly protein 1-like 5 (*NAP1L5*).

In addition, 16 proteins were significantly downregulated in MSA (*p* < 0.05 and log_2_ fold change < −1) (Figure 2A, turquoise dots). These included Orosomucoid 1 (ORM1)-like protein 1 (*ORMDL1*), ORM1-like protein 2 (*ORMDL2*), voltage-dependent calcium channel subunit alpha-2/delta-1 (*CACNA2D1*), caspase-7 (*CASP7*), torsin-1A-interacting protein 1 (*TOR1AIP1*), HAUS (homologous to augmin subunit) augmin-like complex subunit 4 (*HAUS4*), organic solute carrier partner 1 (*OSCP1*), voltage-gated potassium channel subunit Kv7.2 (*KCNQ2*), BEN (BANP, E5R and Nac1) domain-containing protein 5 (*BEND5*), collectin-12 (*COLEC12*), epidermal growth factor receptor (*EGFR*), DNA damage-binding protein 2 (*DDB2*), DENN (differentially expressed in normal and neoplastic cells) domain-containing protein 5B (*DENND5B*), queuosine 5′-phosphate *N*-glycosylase/hydrolase (*C9orf64*), Yip1 domain family member 2 (*YIPF2*) and plakophilin-2 (*PKP2*).

For the 16 downregulated (Figure 2B) and the 25 upregulated (Figure 2C) proteins, the normalised log_2_ intensities for the controls (CTR) and MSA cell lines (MSA) were averaged and displayed as heat maps. The colours of the heat maps depict the mean log_2_ protein intensities, with a brighter colour representing higher protein intensities and a darker colour representing lower protein intensities.

As examples of the calculations of the log_2_ fold changes and *p*-values by unpaired two-tailed *t*-tests, the normalised and averaged log_2_ intensities were visualised as scatter plots, with the averaged log_2_ intensities of one cell line represented as one data point. The two voltage-gated channel subunits *CACNA2D1* (Figure 2D) and *KCNQ2* (Figure 2E) were chosen as examples of functional proteins, *EGFR* (Figure 2F) as a protein with many functions, and *UQCC1* (Figure 2G), *COQ5* (Figure 2H) and *MRPL47* (Figure 2I) as mitochondrial proteins. The protein alpha-synuclein (*SNCA*) (*p* = 0.1415) was additionally displayed as a reference due to the known alterations in MSA (Figure 2J). The log_2_ fold changes were calculated by subtracting the mean log_2_ intensities of the MSA group from the mean log_2_ intensities of the CTR group.

### 3.3. Gene Ontology Analysis

**Figure 3:**
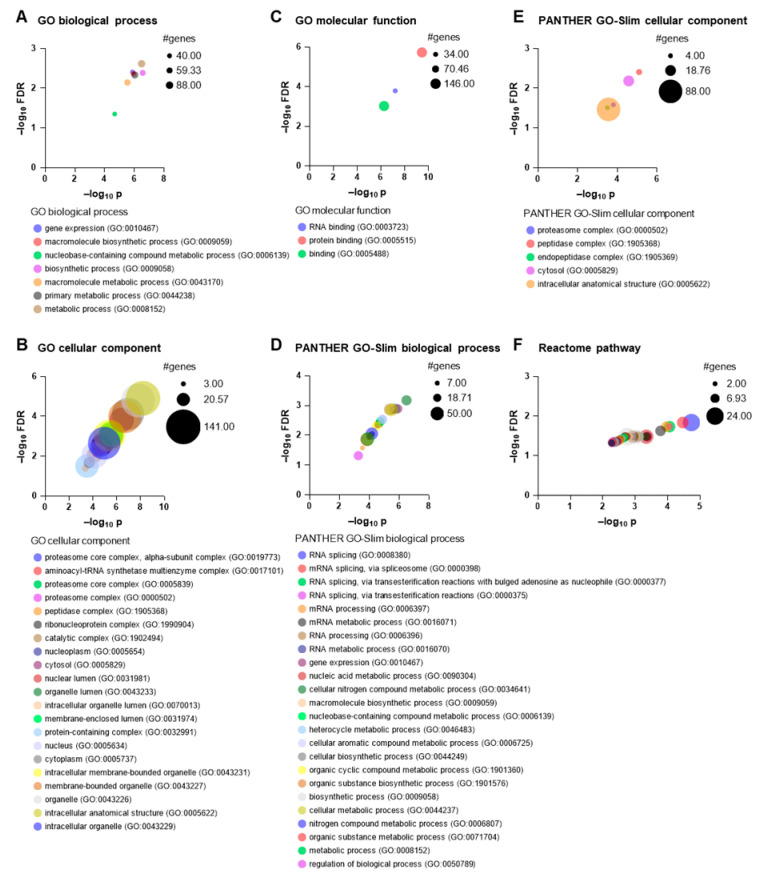
Over-representation analysis of 151 genes using the PANTHER classification system and the Reactome database. Significant protein expressions were examined (*p* < 0.05, unpaired two-tailed *t*-test), derived from three independent differentiations of three CTR cell lines (*n* = 3) and three MSA cell lines (*n* = 3) (see Table S2 for the protein list). For the analysis, the *p*-values (*p*) were calculated by Fisher’s exact test in the PANTHER classification system (**A**–**E**) [27] or the hypergeometric distribution in the Reactome database (**F**) [28], false discovery rate (FDR) was determined according to Benjamini–Hochberg (**A**–**F**). The values were converted to −log_10_ *p*-values and plotted graphically. The bubble size indicates the number (#) of genes found in the Gene Ontology (GO), PANTHER GO-Slim or Reactome pathways. GO terms and the corresponding GO number are listed below the graphs (**A**–**E**). The result of the Reactome over-representation analysis revealed 88 Reactome pathways (*p* < 0.05 and FDR < 0.05) (**F**), in which the reactions can be assigned to the areas of cell cycle, cell death, degradation, axon guidance, neuronal development, cellular response, signalling, translation, viral infection and regulation. A detailed list of the Reactome pathways can be found in the Supplement (Table S3).

For the investigation and categorisation of the different functions of the 151 significant proteins (*p* < 0.05, unpaired two-tailed *t*-test), the corresponding gene names were entered into the PANTHER classification system [27]. They were categorised according to the Gene Ontology (GO) and PANTHER’s own classification. Firstly, the 151 genes were analysed for over-representation using Fisher’s exact test (significance test, *p* < 0.05), which indicates the probability that the number of genes in this category occurred randomly. In addition, the false discovery rate (FDR) was calculated according to Benjamini–Hochberg, which indicates whether the classifications are false positives. With an FDR < 0.05, it can be assumed that up to 95% of the classification is a true assignment [27]. For the 151 genes, seven GO biological processes (Figure 3A), 21 GO cellular components (Figure 3B), three GO molecular functions (Figure 3C), 24 PANTHER GO-Slim biological processes (Figure 3D) and five PANTHER GO-Slim cellular components could be assigned with *p* < 0.05 and FDR < 0.05, whereby four genes (*H1F0*, *C11orf30*, *EEF1E1*-*BLOC1S5*, *EIF2S3L*) were not mapped in the over-representation analysis. The different number of annotations (e.g., 24 or 7 for the biological processes) resulted from the fact that the PANTHER GO-Slim uses the phylogenetic approach with experimental and extrapolated data, while GO considers manual and electronic data [27]. However, common annotations could be found for the biological processes (biosynthetic process (GO:0009058), gene expression (GO:0010467), macromolecule biosynthetic process (GO:0009059), metabolic process (GO:0008152), nucleobase-containing compound metabolic process (GO:0006139)) (Figure 3A,D) and for the cellular components (cytosol (GO:0005829), intracellular anatomical structure (GO:0005622), peptidase complex (GO:1905368) and proteasome complex (GO:0000502)) (Figure 3B,E). Binding was generally specified as a molecular function obtained from the GO annotation (Figure 3C). The over-representation analysis of the 41 significantly upregulated and downregulated genes revealed no significant association (*p* < 0.05 and FDR < 0.05).

**Figure 4:**
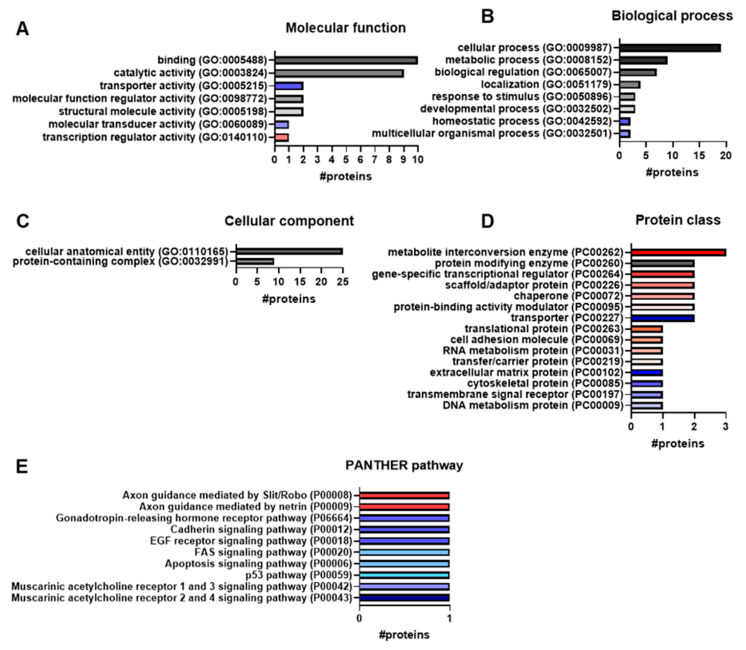
Functional classification of the upregulated and downregulated proteins using PANTHER classification system. Considering the protein expressions from three independent differentiations of three MSA-P cell lines (*n* = 3) and three control cell lines (*n* = 3), 16 significantly downregulated (*p*-value < 0.05, log_2_ fold change < −1) and 25 significantly upregulated proteins (*p*-value < 0.05, log_2_ fold change > 1) were found in MSA-P cell lines (see Table S2 for the protein list). These 41 proteins were classified according to the Gene Ontology (GO) into molecular function (**A**), biological process (**B**) and cellular component (**C**), as well as the PANTHER classification protein class (PC) (**D**) and PANTHER pathway (P) (**E**). The number (#) of classified proteins is stated for each ontology term. Of note, not all proteins could be categorised into these categories, and some proteins were classified into more than one term of one category [27]. The colours of the bars represent the assignment of only downregulated proteins (blue), only upregulated proteins (red) or both types of regulation (grey). For the PANTHER pathways, the same colour was selected to emphasise the allocation of the same protein (**E**).

In addition to the over-representation analysis, a functional classification can be carried out in the PANTHER classification system. For this purpose, the 41 significantly upregulated and downregulated proteins were classified into molecular function (Figure 4A), biological process (Figure 4B) and cellular component (Figure 4C). Not all proteins could be categorised and some proteins fitted more than one term of one ontology [27].

Seven molecular functions, eight biological processes and two cellular components were found as categories. In molecular functions (Figure 4A), most proteins (*RRAGB*, *KCNQ2*, *FABP6*, *PRDM16*, *PQBP1*, *DDB2*, *RNF31*, *EGFR*, *DENND5B* and *HAUS4*) were assigned to binding (GO:0005488). The second most common function was the catalytic activity (GO:0003824), which was attributed to nine proteins (*RRAGB*, *CASP7*, *NMT2*, *SELM*, *RNF31*, *EGFR*, *ENO3*, *PLCXD3* and *COQ5*). Other molecular functions with one to two assigned proteins were structural molecule activity (GO:0005198; *MRPL47* and *COLEC12*), molecular function regulator activity (GO:0098772; *SCG5* and *DENND5B*), transcription regulator activity (GO:0140110; *PRDM16*), transporter activity (GO:0005215; *KCNQ2* and *CACNA2D1*) and molecular transducer activity (GO:0060089; *EGFR*). Notably, the last three ones contained either the upregulated protein *PRDM16* (Figure 4A, red bar) or the downregulated proteins *KCNQ2*, *CACNA2D1* and *EGFR* (Figure 4A, blue bars).

In the category biological process (Figure 4B), 19 proteins (*DCC*, *RRAGB*, *TOR1AIP1*, *CASP7*, *KCNQ2*, *ORMDL1*, *NMT2*, *PQBP1*, *ORMDL2*, *MRPL47*, *CENPV*, *DDB2*, *COLEC12*, *EGFR*, *PKP2*, *ENO3*, *HAUS4*, *PRRC2A* and *C9orf64*) were classified as cellular process (GO:0009987). Nine proteins (*ORMDL1*, *NMT2*, *PQBP1*, *ORMDL2*, *MRPL47*, *DDB2*, *RNF31*, *ENO3* and *C9orf64*) were involved in metabolic processes (GO:0008152). For the biological regulation (GO:0065007), seven proteins (*SCG5*, *RRAGB*, *CASP7*, *KCNQ2*, *PRDM16*, *CENPV* and *EGFR*) were assigned. Other biological processes were localisation (GO:0051179; *KCNQ2*, *FABP6*, *PKP2* and *OSCP1*), response to stimulus (GO:0050896; *RRAGB*, *DDB2* and *EGFR*) and developmental process (GO:0032502; *EGFR*, *PKP2* and *PRRC2A*). For homeostatic processes (GO:0042592; *ORMDL1* and *ORMDL2*) and multicellular organismal processes (GO:0032501; *EGFR* and *PKP2*), only downregulated proteins were classified.

Only two types of cellular components were found (Figure 4C): cellular anatomical entity (GO:0110165) with 25 proteins (*RRAGB*, *TOR1AIP1*, *CASP7*, *KCNQ2*, *ORMDL1*, *FABP6*, *NMT2*, *PRDM16*, *PQBP1*, *ORMDL2*, *MRPL47*, *CENPV*, *SELM*, *DDB2*, *ZC3H4*, *COLEC12*, *EGFR*, *PKP2*, *OSCP1*, *ENO3*, *CACNA2D1*, *HAUS4*, *PLCXD3*, *YIPF2* and *HIRIP3*) and protein-containing complex (GO:0032991) with 9 proteins (*RRAGB*, *KCNQ2*, *ORMDL1*, *ORMDL2*, *MRPL47*, *EGFR*, *ENO3*, *CACNA2D1* and *HAUS4*). For the corresponding protein names, please refer to the protein list in the Supplement (see Table S2).

In addition to the GO terminology, PANTHER also categorises the proteins according to protein classes (PC) (Figure 4D) and PANTHER pathways (P) (Figure 4E). A total of 15 protein classes were identified for 23 proteins (Figure 4D). The proteins *CASP7* and *RNF31* were classified as protein modifying enzymes (PC00260). For the upregulated proteins, nine classes were found, namely scaffold/adaptor protein (PC00226; *COL26A1* and *PQBP1*), chaperone (PC00072; *SCG5* and *UQCC1*), cell adhesion molecule (PC00069; *DCC*), protein-binding activity modulator (PC00095; *RRAGB* and *CYTH3*), transfer/carrier protein (PC00219; *FABP6*), RNA metabolism protein (PC00031; *PRRC2A*), gene-specific transcriptional regulator (PC00264; *PRDM16* and *ZC3H4*), translational protein (PC00263; *MRPL47*) and metabolite interconversion enzyme (PC00262; *NMT2*, *ENO3* and *COQ5*). Six downregulated proteins were assigned to transmembrane signal receptor (PC00197; *EGFR*), transporter (PC00227; *KCNQ2* and *CACNA2D1*), DNA metabolism protein (PC00009; *DDB2*), cytoskeletal protein (PC00085; *PKP2*) and extracellular matrix protein (PC00102; *COLEC12*).

Only six proteins could be assigned to ten PANTHER pathways (Figure 4E). Only the upregulated protein *DCC* was matched to two pathways, axon guidance mediated by Slit/Robo (P00008) and axon guidance mediated by netrin (P00009). Most of the PANTHER pathways were assigned to the downregulated protein *EGFR*: Gonadotropin-releasing hormone receptor pathway (P06664), cadherin signalling pathway (P00012) and EGF receptor signalling pathway (P00018). *CASP7* was also found in two PANTHER pathways, apoptosis signalling pathway (P00006) and FAS signalling pathway (P00020). *CACNA2D1* was associated with the muscarinic acetylcholine receptor 2- and 4-signalling pathway (P00043), *KCNQ2* with muscarinic acetylcholine receptor 1 and 3 signalling pathway (P00042) and *DDB2* with p53 pathway (P00059).

### 3.4. Reactome Analysis

Reactome analysis focuses on the investigation of reactions in biological pathways and processes using the Reactome database. An over-representation analysis with hypergeometric distribution and a Benjamini–Hochberg FDR was performed to determine a probability indicating whether the number of genes in certain Reactome pathways is higher than would be expected by chance [28]. The 151 genes of the respective significant proteins were analysed (*p* < 0.05, unpaired two-tailed *t*-test), whereby 49 were not mapped in the over-representation test. A total of 771 Reactome pathways were found, of which 88 Reactome pathways met the significance criteria (*p* < 0.05 and FDR < 0.05) (Figure 3F). For these, Reactome pathways were identified in the areas of cell cycle, cell death, degradation, axon guidance, neuronal development, cellular response, signalling, translation, viral infection and regulation, which are listed in more detail in the Supplement (see Table S3).

An over-representation analysis was also carried out for the 41 significantly upregulated and downregulated proteins. A total of 237 different Reactome pathways were found for 29 genes and none for 12 genes (upregulated: *OSCP1*, *BEND5*, *YIPF2*; downregulated: *CENPV*, *MTCL1*, *ZER1*, *SCG5*, *SELM*, *HIRIP3*, *PRRC2A*, *PLCXD3*, *NAP1L5*; see Table S2 for the corresponding protein names). For the reduced number of genes, no Reactome pathway met both significance criteria (*p* < 0.05 and FDR < 0.05).

### 3.5. Upstream Regulator Analysis

To identify candidates for the possible origin (upstream regulator) of the normalised and significant proteins (*p* < 0.05, unpaired two-tailed *t*-test), an upstream regulator analysis was performed in Ingenuity Pathway Analysis (IPA). The statistically calculated overlap *p*-value (overlap *p*, one-sided Fisher’s exact test) and the z-score were used to evaluate the results of the upstream regulator analysis. The probability that the experimental dataset did not randomly overlap with the dataset of the Ingenuity Knowledge Base can be specified with an overlap *p* < 0.05. Activation or inhibition, represented by a z-score, was predicted by comparing the examined gene expression with the expected gene expression derived from the literature [29].

The upstream regulator analysis of the 151 significant genes, whereby *SELM* was excluded by IPA, revealed 325 potential candidates that could lead to the observed downregulation or upregulation of proteins. Of these, 320 candidates met the overlap *p* < 0.05, but only 47 received a z-score.

**Figure 5:**
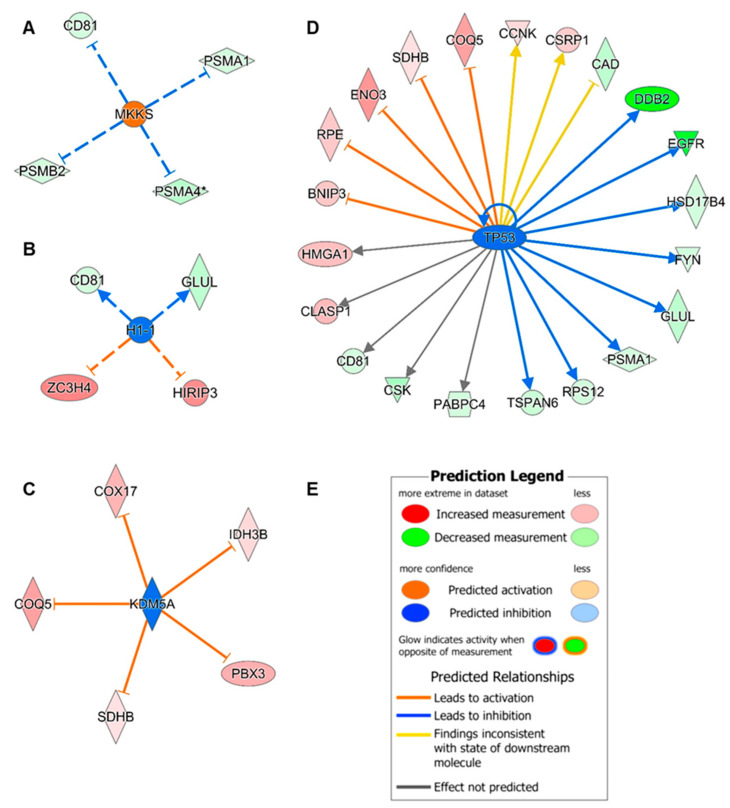
Upstream regulator analysis of significant proteins using Ingenuity Pathway Analysis (IPA, visualisations from IPA, QIAGEN). The data obtained from the proteome analysis of the three CTR cell lines (*n* = 3) and the three MSA cell lines (*n* = 3), each with three independent differentiations, were examined, whereby only the genes determined with the unpaired two-tailed *t*-test with *p* < 0.05 were considered in the upstream analysis. Statistical significance was defined by an overlap *p*-value < 0.05 (one-sided Fisher’s exact test). In addition, z-scores were calculated based on log_2_ fold changes (FC) comparing actual and expected gene expression to predict activation (significant with z-score > 2) or inhibition (significant with z-score < −2) [29]. Using both significance criteria, four molecules were identified as potential upstream regulators, with *MKKS* (**A**) predicted as activated (red), *H1-1* (**B**), *KDM5A* (**C**) and *TP53* (**D**) as inhibited (blue). *TP53* can also self-regulate, as shown by the additional arrow at *TP53*. The predicted relationships from upstream to downstream genes (significant genes from protein expression) were illustrated as dashed lines for indirect relationships or solid lines for direct relationships. Downstream genes’ red and green colours refer to the respective log_2_ FC when comparing protein expressions between MSA and CTR. Colour and arrow type represent the predicted relationships between upstream molecule (activated or inhibited) and downstream molecule (increased or decreased): blue inhibition–I (activated, decreased), blue activation → (inhibited, decreased), orange inhibition–I (inhibited, increased), yellow inhibition–I (inhibited, decreased), yellow activation → (inhibited, increased). Further information is shown in the legend (**E**). *CD81*: CD81 antigen; *PSMA1*: Proteasome subunit alpha type-1; *PSMA4*: Proteasome subunit alpha type-4; *PSMB2*: Proteasome subunit beta type-2; *GLUL*: Glutamine synthetase; *ZC3H4*: Zinc finger C-x8-C-x5-C-x3-H (CCCH) domain-containing protein 4; *HIRIP3*: Histone cell cycle regulator (HIRA)-interacting protein 3; *COX17*: Cytochrome c oxidase copper chaperone; *COQ5*: 2-methoxy-6-polyprenyl-1,4-benzoquinol methylase (mitochondrial); *SDHB*: Succinate dehydrogenase [ubiquinone] iron-sulphur subunit (mitochondrial); *PBX3*: Pre-B-cell leukaemia transcription factor 3; *IDH3B*: Isocitrate dehydrogenase [NAD] subunit beta (mitochondrial); *ENO3*: Beta-enolase; *RPE*: Ribulose-phosphate 3-epimerase; *BNIP3*: B-cell lymphoma 2 (BCL2) interacting protein 3; *HMGA1*: High mobility group protein A1; *CLASP1*: CLIP-associating protein 1; *CSK*: Tyrosine-protein kinase CSK (c-terminal Src kinase); *PABPC4*: Polyadenylate-binding protein 4; *TSPAN6*: Tetraspanin-6; *RPS12*: Ribosomal protein S12; *FYN*: Tyrosine-protein kinase Fyn; *HSD17B4*: Peroxisomal multifunctional enzyme type 2; *EGFR*: Epidermal growth factor receptor; *DDB2*: DNA damage-binding protein 2; *CAD*: Multifunctional protein CAD; *CSRP1*: Cysteine and glycine-rich protein 1; *CCNK*: Cyclin-K.

The molecular chaperone MKKS (Figure 5A), the histone H1.1 (Figure 5B), lysine-specific demethylase 5A (*KDM5A*) (Figure 5C) and cellular tumour antigen p53 (*TP53*) (Figure 5D) were proposed as upstream molecules, which were statistically predicted to be activated (*MKKS*, overlap *p* < 0.05 and z-score > 2) or inhibited (*H1-1*, *KDM5A* and *TP53*, overlap *p* < 0.05 and z-score < −2). For these predictions, 28 of 150 significant genes, shown as downregulated molecules (Figure 5A–D), were linked to the potential upstream molecules, with relationships indicated directly with solid lines or indirectly with dashed lines. Four downstream molecules were observed in more than one prediction. The decreased CD81 antigen (*CD81*) was predicted to be indirectly inhibited by *MKKS*, indirectly activated by *H1-1* and directly linked to *TP53* without directionality, for which the Ingenuity Knowledge Base could not provide any information. *TP53* and the histone *H1-1* could participate in an activation of glutamine synthetase (*GLUL*) leading to the decreased log_2_ FC in MSA cell lines compared to CTR cell lines. *KDM5A* as well as *TP53* were predicted to inhibit 2-methoxy-6-polyprenyl-1,4-benzoquinol methylase (mitochondrial; *COQ5*) and succinate dehydrogenase [ubiquinone] iron-sulphur subunit (mitochondrial; *SDHB*), leading to an increase in both downstream molecules. Yellow relationships of *TP53* to the multifunctional protein CAD (*CAD*), cysteine and glycine-rich protein 1 (*CSRP1*) and cyclin-K (*CCNK*) demonstrate contradictory results in the experimental data and in the literature-based data. In addition to *CD81*, the high mobility group protein A1 (*HMGA1*), CLIP-associating protein 1 (*CLASP1*), tyrosine-protein kinase CSK (c-terminal Src kinase) and polyadenylate-binding protein 4 (*PABPC4*) were also associated with *TP53*, but no activation or inhibition could be predicted.

A repeated upstream regulator analysis was performed with additional cut-offs for log_2_ FC < −1 and log_2_ FC > 1, to predict candidates for the significantly upregulated and downregulated proteins. This filtered the number of genes to 40, resulting in 294 upstream molecules, of which 293 were significant with an overlap *p* < 0.05. For these, no significant activation or inhibition was observed in the z-score calculation. Interestingly, *TP53* was also predicted as an upstream candidate with *COQ5*, epidermal growth factor receptor (*EGFR*), DNA damage-binding protein 2 (*DDB2*) and beta-enolase (*ENO3*) as downstream molecules, but it achieved only an overlap *p* of 0.519 and a z-score of −1.931 (highest score out of four calculated scores). *H1-1* was also found, whereas *MKKS* and *KDM5A* were not.

### 3.6. Potential Biomarker Candidates

Proteome analysis revealed various proteins with different functions that were found in the striatal GABAergic medium spiny neurons of MSA-P cell lines. Of the 41 significantly upregulated and downregulated proteins, 13 with a neuro-specific or neuro-relevant function could be used as potential biomarker candidates (Table 2).

#### 3.6.1. Proteins Associated with Axons and Neurites

Microtubule cross-linking factor 1 is involved in maintaining the initial segment of the neuronal axon [30]. Netrin receptor DCC (Deleted in Colorectal Carcinoma) plays a role in axon outgrowth and guidance [31], which was also demonstrated in the PANTHER pathway result (Figure 4E, axon guidance mediated by Slit/Robo (P00008) and axon guidance mediated by netrin (P00009)). Polyglutamine-binding protein 1 regulates neurite outgrowth [32].

#### 3.6.2. Neuronal Functional Proteins

The postsynaptic protein neurogranin fulfils several synaptic functions, e.g., long-term potentiation [33,34]. Regulation and trafficking of calcium channels depend on the voltage-gated calcium channel subunit alpha-2/delta-1 [35,36]. The voltage-gated potassium channel subunit Kv7.2 is essential for the postsynaptic excitability [37,38].

#### 3.6.3. Neuronal Protection

Glycylpeptide *N*-tetradecanoyltransferase 2 plays a central role in suppressing ferroptosis [39]. Selenoprotein M, which is enriched in the brain, protects the brain from oxidative damage and may also have other functions in calcium regulation [40]. The neuronal Ras-related GTP-binding protein B regulates the mechanistic target of rapamycin complex 1 (mTORC1), which increases resistance to nutrient deprivation [41]. The chaperone protein neuroendocrine protein 7B2 demonstrated an anti-aggregative function by preventing the formation of fibrils from amyloid plaques and alpha-synuclein, which are associated with Alzheimer’s disease and Parkinson’s disease [42].

#### 3.6.4. Myelination

Orosomucoid 1 (ORM1)-like protein 1 and ORM1-like protein 2 are involved in the sphingolipid synthesis, with sphingolipids forming the basis of myelin [43]. The proline-rich coiled coil 2A controls the myelination process and other oligodendrocyte specifications [44].

## 4. Discussion

Biological mechanisms causing or promoting neurodegeneration such as multiple system atrophy (MSA) are not yet fully understood. However, human pluripotent stem cell (hiPSC) models from MSA-P patients can provide new insights into the pathomechanisms. In this study, hiPSCs were differentiated into striatal GABAergic medium spiny neurons, which are the most common cell type in the striatum [45] and are affected by the degeneration that occurs in MSA [4,5].

The proteomes of striatal GABAergic medium spiny neurons derived from cell lines of MSA-P patients obtained a variety of protein types with diverse functions, processes and reactions. In comparison to matched healthy controls, 151 proteins were significantly altered in the protein expressions of MSA cell lines that were tested in over-representation. The results showed that the proteins were over-represented in gene expression, biosynthetic and metabolic processes, were components of various enzyme complexes and located intracellularly or bound to the membrane. Furthermore, the proteins were found in various Reactome pathways including cell cycle, cell death, translation, regulation, degradation, cellular response, signalling, axon guidance, neuronal development and viral infection.

The main neuropathological feature of MSA is the accumulation of aggregated alpha-synuclein, forming cytoplasmic inclusions [5]. As shown in our previous study [7], the neuronal distribution and release of the protein alpha-synuclein was significantly elevated in MSA-P MSNs. In this proteomic study, we found a tendency towards upregulation of alpha-synuclein expression in MSA-P neurons compared to controls (*p* = 0.1415 and log_2_ FC = 1.0668).

In cerebrospinal fluid (CSF) studies of patients with MSA, Parkinson’s disease (PD), progressive supranuclear palsy (PSP), corticobasal syndrome (CBS), dementia with Lewy bodies (DLB) and Alzheimer’s disease (AD), different levels of neurogranin were detected, which were reduced in MSA, PD and PSP and increased in AD compared to controls [21,46]. As a postsynaptic calmodulin-binding protein located in dendritic spines, neurogranin (*NRGN*) fulfils several synaptic functions such as plasticity, regeneration and long-term potentiation [33,34]. Neurogranin is bound to calmodulin at rest. When the calcium concentration changes by influx, calcium ions bind to calmodulin due to changes in affinity, releasing neurogranin and activating the signalling [47,48]. In a study with hippocampal CA1 pyramidal neurons, an overexpression of neurogranin led to increased amplitudes of miniature excitatory postsynaptic currents (mEPSCs) through AMPAR-mediated responses and increased activation of calcium/calmodulin-dependent protein kinase II (CaMKII), resulting in neurogranin-mediated synaptic insertion of glutamate receptor 1 (GluR1) [47]. In contrast, our previous study showed decreased amplitudes in miniature postsynaptic currents of MSA MSNs [7], which could be regulated by neurogranin in an inhibitory, GABA-mediated manner. Calmodulin was also found in both groups, but it did not differ significantly (*p* = 0.6692 and log_2_ fold change = 0.2854).

The two downregulated proteins in MSA cell lines assigned to transporter activity (GO:0005215), the voltage-gated calcium channel subunit alpha-2/delta-1 (*CACNA2D1*) and the voltage-gated potassium channel subunit Kv7.2 (*KCNQ2*), may affect the functional properties of striatal GABAergic medium spiny neurons. The voltage-gated calcium channel subunit alpha-2/delta-1 is one of four other alpha-2/delta subunits that combine to form the auxiliary alpha-2/delta subunit. As an auxiliary alpha-2/delta subunit, it is involved in the regulation of activation and inhibition of the calcium channels Cav1 and Cav2, as well as in the trafficking of Cav2 [35,36]. A reduction in the voltage-gated calcium channel subunit alpha-2/delta-1 could lead to reduced regulation and Cav2 trafficking, which could further explain a reduced frequency of spontaneous calcium transients and a tendency towards decreased gene expression of the Cav2.1 subunit, shown in our previous work [7].

The voltage-gated potassium channel contains the subunits Kv7.2 (*KCNQ2*) and Kv7.3 (*KCNQ3*), which form the neuronal M-current [49], which plays an important role in neuronal excitability, especially in the brain. Under activated condition, the elevated depolarisation threshold is held by a continuous potassium efflux, resulting in hypoexcitability. In contrast, an inhibited or knock-out M-current leads to hyperexcitability [37,38]. A reduction in the voltage-gated potassium channel subunit Kv7.2 could therefore also lead to hyperexcitability due to potentially reduced potassium efflux mechanism. After potassium chloride application, we observed an elevated percentage of responding cells and response amplitude in the calcium imaging of MSA-P MSNs compared to healthy controls; however, the frequencies of spontaneous action potentials and miniature postsynaptic currents were decreased in whole-cell patch-clamp recordings of MSA-P neurons [7].

Three mitochondrial proteins were significantly upregulated in MSA-P neurons, with two proteins having a central role in the mitochondrial respiratory chain. 2-Methoxy-6-polyprenyl-1.4-benzoquinol methylase (*COQ5*) is a precursor in the biosynthesis of the coenzyme Q10 (CoQ10), which supplies electrons to complex III of the mitochondrial respiratory chain. Other proteins involved in the biosynthesis of CoQ10 were found in elevated concentrations in MSA, including decaprenyl diphosphate synthase subunit 2 (*PDSS2*), 4-hydroxy-3-methoxy-5-polyprenylbenzoate decarboxylase (*COQ4*) and aarF domain-containing kinase 3 (*COQ8A*) [13]. The ubiquinol-cytochrome-c reductase complex assembly factor 1 (*UQCC1*) is required for the assembly of the complex III, which was also significantly upregulated in MSA cell lines. Decreased activity of complex II and III was detected in dopaminergic neurons of MSA cell lines [13]. Together with the increased protein expressions, the elevated mitochondrial mass and the reduced activity of the respiratory chain [13], these findings could indicate compensatory mechanisms. As a component of the mitochondrial protein biosynthesis, the large ribosomal subunit protein uL29m (*MRPL47*) was increased not only in striatal GABAergic MSNs but also in the proteome of the cingulate gyrus of sporadic Parkinson’s disease patients (*n* = 3) compared to MSA-P patients (*n* = 3) [6], indicating a higher demand for mitochondrial protein synthesis in both diseases.

As the most frequently occurring protein in PANTHER classification and Reactome pathways, the epidermal growth factor receptor (*EGFR*) was assigned not only to its own pathway (EGF receptor signalling pathway (P00018)), but also to Gonadotropin-releasing hormone receptor pathway (P06664), cadherin signalling pathway (P00012) as well as in the Reactome pathways axon guidance (R-HSA-422475) and nervous system development (R-HSA-9675108), with many non-significant entries also reported. The transmembrane signal protein (PC00197) *EGFR* was also associated with molecular functions such as molecular transducer activity (GO:0060089), binding (GO:0005488), catalytic activity (GO:0003824) and biological processes such as cellular process (GO:0009987), biological regulation (GO:0065007), response to stimulus (GO:0050896), developmental process (GO:0032502) and multicellular organismal process (GO:0032501). This list reflects only a part of the EGFR-mediated processes. In a study with Chinese patients, two polymorphisms of the *EGFR* gene were associated with susceptibility to Parkinson’s disease [50]. A recently published study [51] analysed *EGFR* in the context of alpha-synuclein aggregation in human neuroblastoma cells (SH-SY5Y). It was demonstrated that the phosphorylation of the DnaJ protein homologue 1 (*DNAJB1*) at tyrosine 5, which is carried out by *EGFR*, is essential for the resolution of alpha-synuclein aggregation. Downregulation of *EGFR* resulted in an increase in alpha-synuclein aggregation, which was not reduced by an additional overexpression of *DNAJB1*, indicating the importance of *EGFR* phosphorylation. Furthermore, reduced *EGFR* and *DNAJB1* expression was detected in the brains of PD patients, whereby phosphorylated *DNAJB1* was increased [51]. Accordingly, the reduced expression of *EGFR* in the MSNs of MSA-P cell lines could result in similarly impaired resolution of the aggregation of alpha-synuclein.

To determine whether certain upstream molecules could explain the altered proteome expression, an upstream regulator analysis was performed. The prediction indicated the molecular chaperone MKKS, the histone H1.1, the lysine-specific demethylase 5A (*KDM5A*) and cellular tumour antigen p53 (*TP53*) with significant assignment of activation or inhibition.

The molecular chaperone MKKS (*MKKS*), also known as chaperonin-like *BBS6* protein, can combine with two chaperonin-like BBS proteins (*BBS10* and *BBS12*) and six CCT chaperonin proteins that are required for the assembly of the BBSome complex. The BBSome complex regulates the transport of vesicles to cilia and ciliary signalling [52,53]. An activated *BBS6* could probably induce the BBSome machinery, potentially leading to an increase in vesicle trafficking.

Two subtypes of the histone linker H1, the predicted inhibited histone H1.1, localised on chromosome 6, and the significantly upregulated histone H1.0 (*H1F0* or *H1-0*), localised on chromosome 22, are part of chromatin structure and dynamics, whereby H1.0 is predominantly expressed in terminally differentiated cells [54,55]. During the cell cycle and cell differentiation, the different histone 1 subtypes on chromosome 6 co-regulate each other in a redundant manner, so that compensatory mechanisms could counteract the inhibition of H1.1 [56]. An increase in H1.0 could be a result of an interaction with alpha-synuclein, whereby higher concentrations could induce accumulation and accelerated fibrillation of alpha-synuclein in neurons, which was shown in a study on alpha-synuclein with bovine histone 1 [57].

As a histone demethylase, the lysine-specific demethylase 5A (*KDM5A*, H3K4 demethylase RBP2) is primarily involved in the demethylation of lysine 4 of histone H3 (H3K4me3/me2), thereby deactivating the active state of H3K4me3/2 and suppressing gene expression associated with (neuronal) cell differentiation [58,59]. An inhibition of lysine-specific demethylase 5A might therefore leave the methylated H3K4 in an active state, which could cause overexpression (see Figure 5C). In Parkinson’s disease, the *SNCA* promoter was transcriptionally activated via H3K4me3 and was elevated in the substantia nigra of post-mortem PD brains, resulting in elevated alpha-synuclein expression, which was further investigated in cell models of the neuronal cell line SH-SY5Y and idiopathic PD-iPSC derived dopaminergic neurons. By incorporating the *KDM5A* sequence (also known as *JARID1A*) into the demethylating system targeting the *SNCA* promoter, a reduction in H3K4me3 as well as a decrease in alpha-synuclein expression were achieved in both cell lines [60].

The cellular tumour antigen p53 (*TP53*), also known as phosphoprotein p53 (p53), is a transcription protein regulating many biological processes such as DNA repair, senescence, cell differentiation, stress, apoptosis, cell metabolism and autophagy. Activation and inhibition of p53 functionally regulates many downstream proteins (see Figure 5D) [61,62]. In post-mortem brains, cell and animal models of Parkinson’s disease, elevated p53 values were associated with increased alpha-synuclein expression in the substantia nigra, which is due to regulation of the *SNCA* promoter, but not in other brain regions or related diseases such as MSA [63,64,65].

### Limitations

This proteomic study only reflects the proteome results of striatal GABAergic medium spiny neurons, which limits the observed alterations in protein intensities due to homogenous cell cultures. The inclusion of other relevant cell types, e.g., dopaminergic neurons, oligodendrocytes and astrocytes, in the stem cell model of MSA may provide further insights into the pathomechanisms of the affected cell populations in future studies. In addition, six individual pluripotent stem cell lines derived from three MSA-P patients and three matched healthy controls were analysed, which could also contribute to statistical artefacts in the data. Incorporating more cell lines could minimise individual and statistical effects, but disease-specific cell lines from MSA-patients are hardly available. Apart from the biological limitations, methodological artefacts of the imputed values may also distort the observed changes in protein intensities.

## 5. Conclusions

This study presents the first proteomic analyses of an iPSC model of MSA. Proteome alterations were detected in striatal GABAergic medium spiny neurons from three MSA-P patient-derived cell lines and three matched healthy controls. A comparison of the normalised and averaged protein intensities of the MSA-P group with those of the control group showed a significant change in expression for 151 proteins. By analysing the over-representation, a variety of protein types, molecular functions, biological processes, reactions and cellular components were identified, indicating a multifaceted characteristic of the disease. 25 proteins with at least 2.0-fold upregulation and 16 proteins with less than 0.5-fold downregulation in MSA-P cell lines revealed alterations in neuronal functions such as amplification and attenuation of electrical stimulus transmission, postsynaptic processes, mitochondrial processes, gene regulatory mechanisms, enzymatic processes, apoptosis, signal pathways, etc. As potential upstream regulators, chaperone, histone, histone demethylase and p53 protein were predicted. Since there is currently no disease-modifying therapy for MSA, it is important to gain new insights into the pathomechanisms that may contribute to obtaining possible treatment options.

## Figures and Tables

**Table 1:** Human induced pluripotent stem cell lines from three healthy control subjects and three MSA-P patients. The cell lines CTR1, CTR2 and CTR3 originate from healthy control subjects, while P1, P2 and P3 originate from MSA-P patients.

ID Code	Sex	Age at Biopsy	Origin/Reference
CTR1	M	59	Henkel et al. [7]
CTR2	F	62	StemBANCC consortium SFC084-03-02-01A
CTR3	F	62	Henkel et al. [7]
P1	F	78	Monzio Compagnoni et al. [13]
P2	M	52	Henkel et al. [7]
P3	F	56	Henkel et al. [7]

**Table 2:** Neuro-specific proteins as potential biomarker candidates for multiple system atrophy. Proteins were obtained in the proteome analysis of striatal GABAergic medium spiny neurons (MSNs), which were differentiated from three MSA-P cell lines (*n* = 3) and three CTR cell lines (*n* = 3). The corresponding results of the protein expression are presented in Figure 2. A protein list of the significantly altered protein expressions with *p*-values (unpaired two-tailed *t*-test) and fold changes can be found in the Supplement (Table S2). For the selected proteins proposed here as potential biomarkers, purple arrows indicate those that were upregulated in MSA (log_2_ fold change > 1), and turquoise arrows indicate those that were downregulated in MSA (log_2_ fold change < −1).

Gene Names	Protein Names	MSA vs. CTR	*p*-Value	Fold Change (log_2_)
*MTCL1*	Microtubule cross-linking factor 1	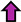	0.0037	2.6503
*NMT2*	Glycylpeptide *N*-tetradecanoyltransferase 2	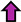	0.0294	1.9344
*SCG5*	Neuroendocrine protein 7B2	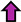	0.0377	1.5578
*SELM*	Selenoprotein M	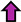	0.0119	1.3475
*RRAGB*	Ras-related GTP-binding protein B	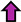	0.0463	1.2842
*PRRC2A*	Proline-rich coiled coil 2A	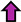	0.0259	1.2435
*DCC*	Netrin receptor DCC (Deleted in Colorectal Carcinoma)	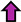	0.0193	1.0619
*PQBP1*	Polyglutamine-binding protein 1	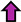	0.0388	1.0494
*NRGN*	Neurogranin	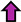	0.0401	1.017
*ORMDL1*	Orosomucoid 1 (ORM1)-like protein 1	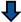	0.0068	−1.071
*ORMDL2*	ORM1-like protein 2	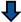	0.0068	−1.071
*CACNA2D1*	Voltage-dependent calcium channel subunit alpha-2/delta-1	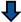	0.0405	−1.1958
*KCNQ2*	Voltage-gated potassium channel subunit Kv7.2	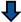	0.0144	−1.4919

## Data Availability

All data are available from the corresponding author by reasonable request.

## Supplementary Materials

The following supporting information can be downloaded at: https://www.mdpi.com/article/10.3390/cells14171394/s1: Table S1: Primer sequences used for RT-qPCR; Table S2: Protein list of significant protein expressions in GABAergic medium spiny neurons of MSA-P cell lines compared to control cell lines; Table S3: Reactome analysis of 151 genes using Reactome database over-representation test.

## References

[1] Jellinger K.A. (2018). Multiple System Atrophy: An Oligodendroglioneural Synucleinopathy. J. Alzheimer’s Dis..

[2] Liu M., Wang Z., Shang H. (2024). Multiple System Atrophy: An Update and Emerging Directions of Biomarkers and Clinical Trials. J. Neurol..

[3] Nandanwar D., Truong D.D. (2024). Multiple System Atrophy: Diagnostic Challenges and a Proposed Diagnostic Algorithm. Clin. Park. Relat. Disord..

[4] Poewe W., Stankovic I., Halliday G., Meissner W.G., Wenning G.K., Pellecchia M.T., Seppi K., Palma J.-A., Kaufmann H. (2022). Multiple System Atrophy. Nat. Rev. Dis. Primers.

[5] Wenning G.K., Stankovic I., Vignatelli L., Fanciulli A., Calandra-Buonaura G., Seppi K., Palma J., Meissner W.G., Krismer F., Berg D. (2022). The Movement Disorder Society Criteria for the Diagnosis of Multiple System Atrophy. Mov. Disord..

[6] Laferrière F., Claverol S., Bezard E., De Giorgi F., Ichas F. (2022). Similar Neuronal Imprint and No Cross-Seeded Fibrils in α-Synuclein Aggregates from MSA and Parkinson’s Disease. NPJ Park. Dis..

[7] Henkel L.M., Kankowski S., Moellenkamp T.M., Smandzich N.J., Schwarz S., Di Fonzo A., Göhring G., Höglinger G., Wegner F. (2023). IPSC-Derived Striatal Medium Spiny Neurons from Patients with Multiple System Atrophy Show Hypoexcitability and Elevated α-Synuclein Release. Cells.

[8] Lin M.T., Beal M.F. (2006). Mitochondrial Dysfunction and Oxidative Stress in Neurodegenerative Diseases. Nature.

[9] Mohamed Yusoff A.A., Mohd Khair S.Z.N. (2025). Unraveling Mitochondrial Dysfunction: Comprehensive Perspectives on Its Impact on Neurodegenerative Diseases. Rev. Neurosci..

[10] Pereira S.L., Grossmann D., Delcambre S., Hermann A., Grünewald A. (2023). Novel Insights into Parkin-Mediated Mitochondrial Dysfunction and Neuroinflammation in Parkinson’s Disease. Curr. Opin. Neurobiol..

[11] Yin F., Sancheti H., Patil I., Cadenas E. (2016). Energy Metabolism and Inflammation in Brain Aging and Alzheimer’s Disease. Free Radic. Biol. Med..

[12] Compta Y., Giraldo D.M., Muñoz E., Antonelli F., Fernández M., Bravo P., Soto M., Cámara A., Torres F., Martí M.J. (2018). Cerebrospinal Fluid Levels of Coenzyme Q10 Are Reduced in Multiple System Atrophy. Park. Relat. Disord..

[13] Monzio Compagnoni G., Kleiner G., Samarani M., Aureli M., Faustini G., Bellucci A., Ronchi D., Bordoni A., Garbellini M., Salani S. (2018). Mitochondrial Dysregulation and Impaired Autophagy in IPSC-Derived Dopaminergic Neurons of Multiple System Atrophy. Stem Cell Rep..

[14] Wan L., Zhu S., Chen Z., Qiu R., Tang B., Jiang H. (2023). Multidimensional Biomarkers for Multiple System Atrophy: An Update and Future Directions. Transl. Neurodegener..

[15] George N.P., Kwon M., Jang Y.E., Kim S.G., Hwang J.S., Lee S.S., Lee G. (2025). Integrative Analysis of Metabolome and Proteome in the Cerebrospinal Fluid of Patients with Multiple System Atrophy. Cells.

[16] Marques T.M., van Rumund A., Kersten I., Bruinsma I.B., Wessels H.J.C.T., Gloerich J., Kaffa C., Esselink R.A.J., Bloem B.R., Kuiperij H.B. (2021). Identification of Cerebrospinal Fluid Biomarkers for Parkinsonism Using a Proteomics Approach. NPJ Park. Dis..

[17] Magdalinou N.K., Noyce A.J., Pinto R., Lindstrom E., Holmén-Larsson J., Holtta M., Blennow K., Morris H.R., Skillbäck T., Warner T.T. (2017). Identification of Candidate Cerebrospinal Fluid Biomarkers in Parkinsonism Using Quantitative Proteomics. Park. Relat. Disord..

[18] Rydbirk R., Østergaard O., Folke J., Hempel C., DellaValle B., Andresen T.L., Løkkegaard A., Hejl A.-M., Bode M., Blaabjerg M. (2022). Brain Proteome Profiling Implicates the Complement and Coagulation Cascade in Multiple System Atrophy Brain Pathology. Cell. Mol. Life Sci..

[19] Dick F., Johanson G.A.S., Tysnes O.-B., Alves G., Dölle C., Tzoulis C. (2025). Brain Proteome Profiling Reveals Common and Divergent Signatures in Parkinson’s Disease, Multiple System Atrophy, and Progressive Supranuclear Palsy. Mol. Neurobiol..

[20] Choi S.G., Tittle T.R., Barot R.R., Betts D.J., Gallagher J.J., Kordower J.H., Chu Y., Killinger B.A. (2025). Proximity Proteomics Reveals Unique and Shared Pathological Features between Multiple System Atrophy and Parkinson’s Disease. Acta Neuropathol. Commun..

[21] Hall S., Janelidze S., Zetterberg H., Brix B., Mattsson N., Surova Y., Blennow K., Hansson O. (2020). Cerebrospinal Fluid Levels of Neurogranin in Parkinsonian Disorders. Mov. Disord..

[22] Laurens B., Constantinescu R., Freeman R., Gerhard A., Jellinger K., Jeromin A., Krismer F., Mollenhauer B., Schlossmacher M.G., Shaw L.M. (2015). Fluid Biomarkers in Multiple System Atrophy: A Review of the MSA Biomarker Initiative. Neurobiol. Dis..

[23] Staege S., Kutschenko A., Baumann H., Glaß H., Henkel L., Gschwendtberger T., Kalmbach N., Klietz M., Hermann A., Lohmann K. (2021). Reduced Expression of GABAA Receptor Alpha2 Subunit Is Associated With Disinhibition of DYT-THAP1 Dystonia Patient-Derived Striatal Medium Spiny Neurons. Front. Cell Dev. Biol..

[24] Stieglitz F., Gerhard R., Hönig R., Giehl K., Pich A. (2022). TcdB of Clostridioides Difficile Mediates RAS-Dependent Necrosis in Epithelial Cells. Int. J. Mol. Sci..

[25] Cox J., Mann M. (2008). MaxQuant Enables High Peptide Identification Rates, Individualized p.p.b.-Range Mass Accuracies and Proteome-Wide Protein Quantification. Nat. Biotechnol..

[26] Tyanova S., Temu T., Sinitcyn P., Carlson A., Hein M.Y., Geiger T., Mann M., Cox J. (2016). The Perseus Computational Platform for Comprehensive Analysis of (Prote)Omics Data. Nat. Methods.

[27] Mi H., Muruganujan A., Huang X., Ebert D., Mills C., Guo X., Thomas P.D. (2019). Protocol Update for Large-Scale Genome and Gene Function Analysis with the PANTHER Classification System (v.14.0). Nat. Protoc..

[28] Rothfels K., Milacic M., Matthews L., Haw R., Sevilla C., Gillespie M., Stephan R., Gong C., Ragueneau E., May B. (2023). Using the Reactome Database. Curr. Protoc..

[29] Krämer A., Green J., Pollard J., Tugendreich S. (2014). Causal Analysis Approaches in Ingenuity Pathway Analysis. Bioinformatics.

[30] Satake T., Yamashita K., Hayashi K., Miyatake S., Tamura-Nakano M., Doi H., Furuta Y., Shioi G., Miura E., Takeo Y.H. (2017). MTCL1 Plays an Essential Role in Maintaining Purkinje Neuron Axon Initial Segment. EMBO J..

[31] Meriane M., Tcherkezian J., Webber C.A., Danek E.I., Triki I., McFarlane S., Bloch-Gallego E., Lamarche-Vane N. (2004). Phosphorylation of DCC by Fyn Mediates Netrin-1 Signaling in Growth Cone Guidance. J. Cell Biol..

[32] Huang X., Cheng S., Han J. (2024). Polyglutamine Binding Protein 1 Regulates Neurite Outgrowth through Recruiting N-WASP. J. Biol. Chem..

[33] Xiang Y., Xin J., Le W., Yang Y. (2020). Neurogranin: A Potential Biomarker of Neurological and Mental Diseases. Front. Aging Neurosci..

[34] Díez-Guerra F.J. (2010). Neurogranin, a Link between Calcium/Calmodulin and Protein Kinase C Signaling in Synaptic Plasticity. IUBMB Life.

[35] Dahimene S., von Elsner L., Holling T., Mattas L.S., Pickard J., Lessel D., Pilch K.S., Kadurin I., Pratt W.S., Zhulin I.B. (2022). Biallelic CACNA2D1 Loss-of-Function Variants Cause Early-Onset Developmental Epileptic Encephalopathy. Brain.

[36] Dolphin A.C., Lee A. (2020). Presynaptic Calcium Channels: Specialized Control of Synaptic Neurotransmitter Release. Nat. Rev. Neurosci..

[37] Greene D.L., Hoshi N. (2017). Modulation of Kv7 Channels and Excitability in the Brain. Cell. Mol. Life Sci..

[38] Hou B., Varghese N., Soh H., Santaniello S., Tzingounis A.V. (2021). Loss of KCNQ2 or KCNQ3 Leads to Multifocal Time-Varying Activity in the Neonatal Forebrain Ex Vivo. eNeuro.

[39] Liu N., Wu W.-L., Wan X.-R., Wang J., Huang J.-N., Jiang Y.-Y., Sheng Y.-C., Wu J.-C., Liang Z.-Q., Qin Z.-H. (2024). Regulation of FSP1 Myristoylation by NADPH: A Novel Mechanism for Ferroptosis Inhibition. Redox Biol..

[40] Reeves M.A., Bellinger F.P., Berry M.J. (2010). The Neuroprotective Functions of Selenoprotein M and Its Role in Cytosolic Calcium Regulation. Antioxid. Redox Signal.

[41] Figlia G., Müller S., Hagenston A.M., Kleber S., Roiuk M., Quast J.-P., ten Bosch N., Carvajal Ibañez D., Mauceri D., Martin-Villalba A. (2022). Brain-Enriched RagB Isoforms Regulate the Dynamics of MTORC1 Activity through GATOR1 Inhibition. Nat. Cell Biol..

[42] Helwig M., Hoshino A., Berridge C., Lee S.-N., Lorenzen N., Otzen D.E., Eriksen J.L., Lindberg I. (2013). The Neuroendocrine Protein 7B2 Suppresses the Aggregation of Neurodegenerative Disease-Related Proteins. J. Biol. Chem..

[43] Clarke B.A., Majumder S., Zhu H., Lee Y.T., Kono M., Li C., Khanna C., Blain H., Schwartz R., Huso V.L. (2019). The Ormdl Genes Regulate the Sphingolipid Synthesis Pathway to Ensure Proper Myelination and Neurologic Function in Mice. Elife.

[44] Wu R., Li A., Sun B., Sun J.-G., Zhang J., Zhang T., Chen Y., Xiao Y., Gao Y., Zhang Q. (2019). A Novel M6A Reader Prrc2a Controls Oligodendroglial Specification and Myelination. Cell Res..

[45] Kreitzer A.C., Malenka R.C. (2008). Striatal Plasticity and Basal Ganglia Circuit Function. Neuron.

[46] Nilsson J., Constantinescu J., Nellgård B., Jakobsson P., Brum W.S., Gobom J., Forsgren L., Dalla K., Constantinescu R., Zetterberg H. (2023). Cerebrospinal Fluid Biomarkers of Synaptic Dysfunction Are Altered in Parkinson’s Disease and Related Disorders. Mov. Disord..

[47] Zhong L., Cherry T., Bies C.E., Florence M.A., Gerges N.Z. (2009). Neurogranin Enhances Synaptic Strength through Its Interaction with Calmodulin. EMBO J..

[48] Hoffman L., Chandrasekar A., Wang X., Putkey J.A., Waxham M.N. (2014). Neurogranin Alters the Structure and Calcium Binding Properties of Calmodulin. J. Biol. Chem..

[49] Wang H. (1998). KCNQ2 and KCNQ3 Potassium Channel Subunits: Molecular Correlates of the M-Channel. Science (1979).

[50] Jin J., Xue L., Bai X., Zhang X., Tian Q., Xie A. (2020). Association between Epidermal Growth Factor Receptor Gene Polymorphisms and Susceptibility to Parkinson’s Disease. Neurosci. Lett..

[51] Huang Y.-Y., Lin S.-J., Chiang W.-Y., Chang Y.-T., Yang C.-C., Liao C.-Y., Chang Y.-L., Lin C.-H., Teng S.-C. (2025). EGFR Phosphorylates DNAJB1 to Suppress α-Synuclein Aggregation in Parkinson’s Disease. NPJ Park. Dis..

[52] Seo S., Baye L.M., Schulz N.P., Beck J.S., Zhang Q., Slusarski D.C., Sheffield V.C. (2010). BBS6, BBS10, and BBS12 Form a Complex with CCT/TRiC Family Chaperonins and Mediate BBSome Assembly. Proc. Natl. Acad. Sci. USA.

[53] Tian X., Zhao H., Zhou J. (2023). Organization, Functions, and Mechanisms of the BBSome in Development, Ciliopathies, and Beyond. Elife.

[54] Izzo A., Schneider R. (2016). The Role of Linker Histone H1 Modifications in the Regulation of Gene Expression and Chromatin Dynamics. Biochim. Biophys. Acta (BBA)-Gene Regul. Mech..

[55] Albig W., Drabent B., Kunz J., Kalff-Suske M., Grzeschik K.-H., Doenecke D. (1993). All Known Human H1 Histone Genes Except the H10 Gene Are Clustered on Chromosome 6. Genomics.

[56] Ponte I., Andrés M., Jordan A., Roque A. (2021). Towards Understanding the Regulation of Histone H1 Somatic Subtypes with OMICs. J. Mol. Biol..

[57] Goers J., Manning-Bog A.B., McCormack A.L., Millett I.S., Doniach S., Di Monte D.A., Uversky V.N., Fink A.L. (2003). Nuclear Localization of α-Synuclein and Its Interaction with Histones. Biochemistry.

[58] Tu S., Teng Y.-C., Yuan C., Wu Y.-T., Chan M.-Y., Cheng A.-N., Lin P.-H., Juan L.-J., Tsai M.-D. (2008). The ARID Domain of the H3K4 Demethylase RBP2 Binds to a DNA CCGCCC Motif. Nat. Struct. Mol. Biol..

[59] Wang H., Guo B., Guo X. (2024). Histone Demethylases in Neurodevelopment and Neurodegenerative Diseases. Int. J. Neurosci..

[60] Guhathakurta S., Kim J., Adams L., Basu S., Song M.K., Adler E., Je G., Fiadeiro M.B., Kim Y. (2021). Targeted Attenuation of Elevated Histone Marks at SNCA Alleviates A-synuclein in Parkinson’s Disease. EMBO Mol. Med..

[61] Hernández Borrero L.J., El-Deiry W.S. (2021). Tumor Suppressor P53: Biology, Signaling Pathways, and Therapeutic Targeting. Biochim. Biophys. Acta (BBA)-Rev. Cancer.

[62] Wang H., Guo M., Wei H., Chen Y. (2023). Targeting P53 Pathways: Mechanisms, Structures and Advances in Therapy. Signal Transduct. Target. Ther..

[63] Duplan E., Giordano C., Checler F., Alves da Costa C. (2016). Direct α-Synuclein Promoter Transactivation by the Tumor Suppressor P53. Mol. Neurodegener..

[64] López K.L.R., Simpson J.E., Watson L.C., Mortiboys H., Hautbergue G.M., Bandmann O., Highley J.R. (2019). TIGAR Inclusion Pathology Is Specific for Lewy Body Diseases. Brain Res..

[65] Probst-Cousin S., Rickert C.H., Schmid K.W., Gullota F. (1998). Cell Death Mechanisms in Multiple System Atrophy. J. Neuropathol. Exp. Neurol..

