# Supplementary material for: Proteomics of Patient-Derived Striatal Medium Spiny Neurons in Multiple System Atrophy
Source: Cells. 2025 Sep 6;14(17):1394. doi: 10.3390/cells14171394 (PMC12428458; doi:10.3390/cells14171394)
Supplement: Supplementary file 1 [file cells-14-01394-s001.zip › cells-3845743-supplementary.pdf]

Supplementary materials

Table S1. Primer sequences used for RT-qPCR [7,23]. GABAergic (GAD67, glutamic acid decarboxylase; FOXG1, forkhead box protein G1), striatal (CTIP2, COUP TF-1 interacting protein 2), neuronal (TUJ1, beta 3 tubulin; MAP2, microtubule-associated protein 2; FOXP1, forkhead box protein P1) and reference genes (B2M, beta-2 microglobulin; GAPDH, glyceraldehyde-3-phosphate dehydrogenase; ACTB, beta-actin) are listed.

Target		Sequence (5′-3′)
GAD67	Forward	AGATCAACAAATGCCTGGAACTGGC
	Reverse	GAGCCACCTTGTGTAGCTTTTCCC
FOXG1	Forward	AGCAGCACTTTGAGTTACAACGGC
	Reverse	CTGAGTCAACACGGAGCTGTAGGG
CTIP2	Forward	CTCCGAGCTCAGGAAAGTGTC
	Reverse	TCATCTTTACCTGCAATGTTCTCC
TUJ1	Forward	AGTGATGAGCATGGCATCGACCC
	Reverse	GGCACGTACTTGTGAGAAGAGGC
MAP2	Forward	CAGGCAAAGGACAAAGTCTCTGACG
	Reverse	CGCCGAGGAGGGAGAATGGAGG
FOXP1	Forward	CCACGTGGAAGAATGCAGTGCG
	Reverse	GCATTGAGAGGTGTGCAGTAGGC
B2M	Forward	TGCCTGCCGTGTGAACCATGT
	Reverse	TGCGGAATCTTCAAACCTCCATGA
GAPDH	Forward	AGCCACATCGCTCAGACACCAT
	Reverse	CAGGCGCCCAATACGACCAAAT
ACTB	Forward	CATGTACGTTGCTATCCAGGC
	Reverse	CTCCTTAATGTCACGCACGAT

Table S2. Protein list of significant protein expressions in GABAergic medium spiny neurons of MSA-P cell lines compared to control cell lines. Protein expressions derived from three independent differentiations of three MSA-P cell lines (*n*=3) and three control cell lines (*n*=3) and grouped accordingly. Normalised and averaged $\log_2$ protein intensities were compared between MSA and CTR groups. Fold changes represent observed expression shift of MSA cell lines. 151 proteins are listed with gene names, *p*-values (unpaired two-tailed t-test) and $\log_2$ fold changes.

Gene Names	Protein Names	*P*-value	Fold Change ($\log_2$)
FABP6	Gastrotropin	0.0407	2.9058
CENPV	Centromere protein V	0.0394	2.8798
MTCL1	Microtubule cross-linking factor 1	0.0037	2.6503
PRDM16	Histone-lysine N-methyltransferase PR-domain containing 16	0.0125	2.5821
ZER1	Protein zer-1 homolog	0.0384	2.1870
NMT2	Glycylpeptide N-tetradecanoyltransferase 2	0.0294	1.9344
CYTH3	Cytohesin-3	0.0095	1.6908
SCG5	Neuroendocrine protein 7B2	0.0377	1.5578
ZC3H4	Zinc finger C-x8-C-x5-C-x3-H (CCCH) domain-containing protein 4	0.0465	1.4389
SELM	Selenoprotein M	0.0119	1.3475
HIRIP3	Histone cell cycle regulator (HIRA)-interacting protein 3	0.0233	1.3316
RRAGB	Ras-related GTP-binding protein B	0.0463	1.2842
UQCC1	Ubiquinol-cytochrome-c reductase complex assembly factor 1	0.0332	1.2776
PRRC2A	Proline rich coiled coil 2A	0.0259	1.2435

ENO3	Beta-enolase	0.0274	1.2432
RNF31	E3 ubiquitin-protein ligase RNF31	0.0478	1.2097
PLCXD3	Phosphatidylinositol specific phospholipase C X domain containing 3	0.0275	1.2010
COL26A1	Collagen alpha-1(XXVI) chain	0.0276	1.1974
H1F0	Histone H1.0	0.0259	1.1691
COQ5	2-methoxy-6-polyprenyl-1.4-benzoquinol methylase. mitochondrial	0.0042	1.1202
MRPL47	Large ribosomal subunit protein uL29m	0.0348	1.1158
DCC	Netrin receptor DCC (Deleted in Colorectal Carcinoma)	0.0193	1.0619
PQBP1	Polyglutamine-binding protein 1	0.0388	1.0494
NRGN	Neurogranin	0.0401	1.0170
NAP1L5	Nucleosome assembly protein 1-like 5	0.0498	1.0020
ICA1L	Islet cell autoantigen 1-like protein	0.0244	0.9781
PIR	Pirin	0.0366	0.9734
PBX3	Pre-B-cell leukemia transcription factor 3	0.0047	0.9570
PFKFB2	6-phosphofructo-2-kinase/fructose-2.6-bisphosphatase 2	0.0089	0.9504
GRIPAP1	GRIP1-associated protein 1	0.0255	0.9286
ATPIF1	ATPase inhibitor. mitochondrial	0.0189	0.9156
SP3	Transcription factor Sp3	0.0210	0.8875
COX17	Cytochrome c oxidase copper chaperone	0.0283	0.8735
CLASP1	CLIP-associating protein 1	0.0146	0.8231
TAF9	Transcription initiation factor TFIID subunit 9	0.0288	0.8208
TAF9B	Transcription initiation factor TFIID subunit 9B	0.0288	0.8208
POMGNT2	Protein O-linked-mannose beta-1.4-N-acetylglucosaminyltransferase 2	0.0447	0.7651
CRABP2	Cellular retinoic acid-binding protein 2	0.0339	0.7635
DNAJC17	DnaJ homolog subfamily C member 17	0.0005	0.7543
TMEM128	Transmembrane protein 128	0.0467	0.7484
SLC8A2	Sodium/calcium exchanger 2	0.0387	0.7483
DPM3	Dolichol-phosphate mannosyltransferase subunit 3	0.0130	0.7412
HMGA2	High mobility group AT-Hook 2	0.0333	0.6855
BNIP3	B-cell lymphoma 2 (BCL2) interacting protein 3	0.0392	0.6695
RPE	Ribulose-phosphate 3-epimerase	0.0239	0.6581
PRRT1	Proline-rich transmembrane protein 1	0.0052	0.6394
RPL31	60S ribosomal protein L31	0.0453	0.6336
DCP1A	mRNA-decapping enzyme 1A	0.0139	0.6242
BRD2	Bromodomain-containing protein 2	0.0391	0.6216
CSRP1	Cysteine and glycine-rich protein 1	0.0344	0.6158
SMG8	SMG8 nonsense mediated mRNA decay factor	0.0068	0.5950
PBDC1	Polysaccharide biosynthesis domain containing 1	0.0409	0.5416
BANF1	Barrier-to-autointegration factor	0.0487	0.5393
ZNF428	Zinc finger protein 428	0.0225	0.5351
DNER	Delta and Notch-like epidermal growth factor-related receptor	0.0452	0.5182
FHL3	Four and a half LIM domains protein 3	0.0282	0.5165
SLU7	Pre-mRNA-splicing factor SLU7	0.0206	0.5067
LYRM7	Complex III assembly factor LYRM7	0.0438	0.4946
ZFTRAF1	Zinc finger TRAF-type-containing protein 1	0.0245	0.4886
TBCA	Tubulin-specific chaperone A	0.0108	0.4655
IK	Cytokine IK	0.0305	0.4617
ZNF362	Zinc finger protein 362	0.0254	0.4568
CCNK	Cyclin-K	0.0127	0.4457
RPS7	40S ribosomal protein S7	0.0215	0.4435
IDH3B	Isocitrate dehydrogenase [NAD] subunit beta. mitochondrial	0.0281	0.4400

SCAF8	SR-related CTD associated factor 8	0.0277	0.4361
EMSY;C11orf30	BRCA2-interacting transcriptional repressor EMSY	0.0418	0.4294
PRRC2C	Proline rich coiled coil 2C	0.0053	0.4023
BAG4	BAG family molecular chaperone regulator 4	0.0281	0.3652
NCKIPSD	NCK-interacting protein with SH3 domain	0.0146	0.3549
RBM5	RNA-binding protein 5	0.0290	0.3450
CLIP1	CAP-Gly domain-containing linker protein 1	0.0442	0.3217
ASF1A	Histone chaperone ASF1A	0.0198	0.3127
RPL23A	60S ribosomal protein L23a	0.0306	0.3116
RBM6	RNA-binding protein 6	0.0417	0.2968
HAGH	Hydroxyacylglutathione hydrolase. mitochondrial	0.0042	0.2791
UHRF1BP1	UHRF1-binding protein 1	0.0368	0.2530
POLDIP3	Polymerase delta-interacting protein 3	0.0047	0.2393
SDHB	Succinate dehydrogenase [ubiquinone] iron-sulfur subunit. mitochondrial	0.0041	0.2331
DNPH1	2-deoxynucleoside 5-phosphate N-hydrolase 1	0.0187	0.2169
BPNT1	3(2).5-bisphosphate nucleotidase 1	0.0052	0.2137
RPS12	40S ribosomal protein S12	0.0245	–0.1180
SH3GLB1	Endophilin-B1	0.0258	–0.1300
TPR	Nucleoprotein TPR	0.0041	–0.1370
AIMP1	Aminoacyl tRNA synthase complex-interacting multifunctional protein 1	0.0329	–0.1706
PABPC4	Polyadenylate-binding protein 4	0.0187	–0.1854
PSMA1	Proteasome subunit alpha type-1	0.0254	–0.2046
PCBP1	Poly(rC)-binding protein 1	0.0056	–0.2339
FYN	Tyrosine-protein kinase Fyn	0.0064	–0.2350
TXNL1	Thioredoxin-like protein 1	0.0223	–0.2455
DARS	Aspartate--tRNA ligase. cytoplasmic	0.0489	–0.2480
CD81	CD81 antigen	0.0241	–0.2621
PSMB2	Proteasome subunit beta type-2	0.0021	–0.2682
HSD17B4	Peroxisomal multifunctional enzyme type 2	0.0432	–0.2687
DPP3	Dipeptidyl peptidase 3	0.0432	–0.2690
TMX1	Thioredoxin-related transmembrane protein 1	0.0479	–0.2710
PSMA6	Proteasome subunit alpha type-6	0.0417	–0.2841
CCAR2	Cell cycle and apoptosis regulator protein 2	0.0442	–0.3092
EMC3	ER membrane protein complex subunit 3	0.0113	–0.3108
CERS1	Ceramide synthase 1	0.0049	–0.3174
FADD	FAS-associated death domain protein	0.0310	–0.3229
POU2F1	POU domain. class 2. transcription factor 1	0.0019	–0.3619
POU2F3	POU domain. class 2. transcription factor 3	0.0019	–0.3619
EEF1E1	Eukaryotic translation elongation factor 1 epsilon-1	0.0115	–0.3659
EEF1E1-BLOC1S5	EEF1E1-BLOC1S5 readthrough (NMD candidate)	0.0115	–0.3659
SBDS	Ribosome maturation protein SBDS	0.0282	–0.3714
MAP3K7	Transforming growth factor-beta-activated kinase 1	0.0373	–0.3862
DUSP3	Dual specificity protein phosphatase 3	0.0417	–0.3870
LLPH	LLP homolog, long-term synaptic facilitation factor	0.0028	–0.3929
EIF2S3; EIF2S3L	Eukaryotic translation initiation factor 2 subunit 3;<br>Putative eukaryotic translation initiation factor 2 subunit 3-like protein	0.0500	–0.4024
TSPAN6	Tetraspanin-6	0.0458	–0.4065
SYPL1	Synaptophysin-like protein 1	0.0075	–0.4124
ATG3	Ubiquitin-like-conjugating enzyme ATG3	0.0186	–0.4278
UMAD1	UBAP1-MVB12-associated (UMA)-domain containing protein 1	0.0232	–0.4605

CAD	Multifunctional protein CAD	0.0230	–0.4735
GLUL	Glutamine synthetase	0.0052	–0.4784
UTP18	U3 small nucleolar RNA-associated protein 18 homolog	0.0405	–0.4965
YIF1B	Yip1 interacting factor homolog B, membrane trafficking protein	0.0174	–0.5107
CDYL	Chromodomain Y-like protein	0.0317	–0.5125
BZW1	Basic leucine zipper and W2 domain-containing protein 1	0.0257	–0.5208
TRMT61A	tRNA (adenine(58)-N(1))-methyltransferase catalytic subunit TRMT61A	0.0303	–0.5398
PSMA4	Proteasome subunit beta type	0.0187	–0.5400
RBMX2	RNA-binding motif protein. X-linked 2	0.0366	–0.5618
KIF13B	Kinesin-like protein KIF13B	0.0098	–0.5632
AAR2	AAR2 splicing factor homolog	0.0470	–0.5804
THG1L	Probable tRNA(His) guanylyltransferase	0.0306	–0.6128
RP2	RP2 activator of ARL3 GTPase	0.0481	–0.6317
CSK	Tyrosine-protein kinase CSK	0.0068	–0.6353
C10orf35	Uncharacterized protein C10orf35	0.0428	–0.6623
TUBA4A	Tubulin alpha 4a	0.0331	–0.6720
IGLON5	IgLON family member 5	0.0019	–0.6925
GOLIM4	Golgi integral membrane protein 4	0.0340	–0.8264
SSR3	Translocon-associated protein subunit gamma	0.0357	–0.8473
MMTAG2	Multiple myeloma tumor-associated protein 2	0.0373	–0.8591
GPRIN3	G protein-regulated inducer of neurite outgrowth 3	0.0288	–0.9792
ORMDL1	Orosomucoid 1 (ORM1)-like protein 1	0.0068	–1.0710
ORMDL2	ORM1-like protein 2	0.0068	–1.0710
CACNA2D1	Voltage-dependent calcium channel subunit alpha-2/delta-1	0.0405	–1.1958
CASP7	Caspase-7	0.0484	–1.2115
TOR1AIP1	Torsin-1A-interacting protein 1	0.0220	–1.2466
HAUS4	HAUS (homologous to augmin subunit) augmin-like complex subunit 4	0.0183	–1.3507
OSCP1	Organic solute carrier partner 1	0.0295	–1.4046
KCNQ2	Voltage-gated potassium channel subunit Kv7.2	0.0144	–1.4919
BEND5	BEN (BANP, E5R and Nac1) domain-containing protein 5	0.0031	–1.8533
COLEC12	Collectin-12	0.0043	–1.9175
EGFR	Epidermal growth factor receptor	0.0266	–1.9632
DDB2	DNA damage-binding protein 2	0.0292	–2.2595
DENND5B	DENN (differentially expressed in normal and neoplastic cells) domain-containing protein 5B	0.0498	–2.3834
C9orf64	Queuosine 5'-phosphate N-glycosylase/hydrolase	0.0064	–2.4078
YIPF2	Yip1 domain family member 2	0.0117	–2.8732
PKP2	Plakophilin-2	0.0259	–2.9595

Table S3. Reactome analysis of 151 genes using Reactome database over-representation test. Protein expressions derived from three independent differentiations of three MSA-P cell lines (*n*=3) and three control cell lines (*n*=3) were used in the over-representation analysis of the Reactome database. *P*-values were calculated by the hypergeometric distribution, false discovery rate (FDR) according to Benjamini-Hochberg [28]. The 88 Reactome pathways with pathway identifiers for which both significance criteria ($p < 0.05$ and FDR < 0.05) were met are listed below. For each Reactome pathway, the number of assigned genes is indicated with #genes.

Pathway Identifier	Pathway Name	#genes	*P*-value	FDR
R-HSA-8953854	Metabolism of RNA	24	1.84E–05	0.0146
R-HSA-376176	Signaling by ROBO receptors	11	3.41E–05	0.0146
R-HSA-162906	HIV Infection	11	8.77E–05	0.0186
R-HSA-9912633	Antigen processing: Ub, ATP-independent proteasomal degradation	4	8.83E–05	0.0186
R-HSA-9010553	Regulation of expression of SLITs and ROBOs	9	1.09E–04	0.0186
R-HSA-109581	Apoptosis	9	1.66E–04	0.0238
R-HSA-9766229	Degradation of CDH1	5	4.19E–04	0.0332
R-HSA-927802	Nonsense-Mediated Decay (NMD)	7	4.37E–04	0.0332
R-HSA-975957	Nonsense Mediated Decay (NMD) enhanced by the Exon Junction Complex (EJC)	7	4.37E–04	0.0332
R-HSA-422475	Axon guidance	16	4.54E–04	0.0332
R-HSA-1169091	Activation of NF-kappaB in B cells	5	4.89E–04	0.0332
R-HSA-211733	Regulation of activated PAK-2p34 by proteasome mediated degradation	4	7.45E–04	0.0332
R-HSA-9764561	Regulation of CDH1 Function	5	7.54E–04	0.0332
R-HSA-162909	Host Interactions of HIV factors	7	8.15E–04	0.0332
R-HSA-350562	Regulation of ornithine decarboxylase (ODC)	4	8.22E–04	0.0332
R-HSA-5357801	Programmed Cell Death	9	8.23E–04	0.0332
R-HSA-2559584	Formation of Senescence-Associated Heterochromatin Foci (SAHF)	3	8.79E–04	0.0332
R-HSA-2408522	Selenoamino acid metabolism	8	8.81E–04	0.0332
R-HSA-9675108	Nervous system development	16	8.87E–04	0.0332
R-HSA-180534	Vpu mediated degradation of CD4	4	9.92E–04	0.0332
R-HSA-8852276	The role of GTSE1 in G2/M progression after G2 checkpoint	5	1.05E–03	0.0332
R-HSA-75815	Ubiquitin-dependent degradation of Cyclin D	4	1.09E–03	0.0332
R-HSA-349425	Autodegradation of the E3 ubiquitin ligase COP1	4	1.09E–03	0.0332
R-HSA-8854050	FBXL7 down-regulates AURKA during mitotic entry and in early mitosis	4	1.19E–03	0.0332
R-HSA-174113	SCF-beta-TrCP mediated degradation of Emi1	4	1.19E–03	0.0332
R-HSA-169911	Regulation of Apoptosis	4	1.19E–03	0.0332
R-HSA-72766	Translation	12	1.21E–03	0.0332
R-HSA-450408	AUF1 (hnRNP D0) binds and destabilizes mRNA	4	1.29E–03	0.0332
R-HSA-180585	Vif-mediated degradation of APOBEC3G	4	1.29E–03	0.0332
R-HSA-4641258	Degradation of DVL	4	1.41E–03	0.0332
R-HSA-4641257	Degradation of AXIN	4	1.41E–03	0.0332
R-HSA-9762114	GSK3B and BTRC:CUL1-mediated-degradation of NFE2L2	4	1.41E–03	0.0332
R-HSA-1236978	Cross-presentation of soluble exogenous antigens (endosomes)	4	1.65E–03	0.0332
R-HSA-69613	p53-Independent G1/S DNA Damage Checkpoint	4	1.65E–03	0.0332
R-HSA-69601	Ubiquitin-Mediated Degradation of Phosphorylated Cdc25A	4	1.65E–03	0.0332
R-HSA-69541	Stabilization of p53	4	1.65E–03	0.0332
R-HSA-9633012	Response of EIF2AK4 (GCN2) to amino acid deficiency	6	1.66E–03	0.0332
R-HSA-1168372	Downstream signaling events of B Cell Receptor (BCR)	5	1.68E–03	0.0332
R-HSA-8953897	Cellular responses to stimuli	24	1.76E–03	0.0332
R-HSA-9604323	Negative regulation of NOTCH4 signaling	4	1.78E–03	0.0332
R-HSA-8941858	Regulation of RUNX3 expression and activity	4	1.78E–03	0.0332
R-HSA-450531	Regulation of mRNA stability by proteins that bind AU-rich elements	5	1.87E–03	0.0332
R-HSA-5362768	Hh mutants are degraded by ERAD	4	1.93E–03	0.0332

R-HSA-5676590	NIK-->noncanonical NF-kB signaling	4	1.93E–03	0.0332
R-HSA-1799339	SRP-dependent cotranslational protein targeting to membrane	6	1.97E–03	0.0332
R-HSA-5660489	MTF1 activates gene expression	2	1.99E–03	0.0332
R-HSA-72706	GTP hydrolysis and joining of the 60S ribosomal subunit	6	2.06E–03	0.0332
R-HSA-156827	L13a-mediated translational silencing of Ceruloplasmin expression	6	2.06E–03	0.0332
R-HSA-9932298	Degradation of CRY and PER proteins	4	2.07E–03	0.0332
R-HSA-5610780	Degradation of GLI1 by the proteasome	4	2.07E–03	0.0332
R-HSA-5610785	GLI3 is processed to GLI3R by the proteasome	4	2.07E–03	0.0332
R-HSA-5610783	Degradation of GLI2 by the proteasome	4	2.07E–03	0.0332
R-HSA-202403	TCR signaling	6	2.23E–03	0.0357
R-HSA-5387390	Hh mutants abrogate ligand secretion	4	2.39E–03	0.0359
R-HSA-9907900	Proteasome assembly	4	2.57E–03	0.0382
R-HSA-187577	SCF(Skp2)-mediated degradation of p27/p21	4	2.57E–03	0.0382
R-HSA-4608870	Asymmetric localization of PCP proteins	4	2.75E–03	0.0382
R-HSA-5678895	Defective CFTR causes cystic fibrosis	4	2.75E–03	0.0382
R-HSA-5607761	Dectin-1 mediated noncanonical NF-kB signaling	4	2.75E–03	0.0382
R-HSA-9824272	Somitogenesis	4	2.75E–03	0.0382
R-HSA-174084	Autodegradation of Cdh1 by Cdh1:APC/C	4	2.94E–03	0.0382
R-HSA-72737	Cap-dependent Translation Initiation	6	3.05E–03	0.0396
R-HSA-72613	Eukaryotic Translation Initiation	6	3.05E–03	0.0396
R-HSA-174154	APC/C:Cdc20 mediated degradation of Securin	4	3.13E–03	0.0407
R-HSA-9711097	Cellular response to starvation	7	3.19E–03	0.0415
R-HSA-69580	p53-Dependent G1/S DNA damage checkpoint	4	3.55E–03	0.0427
R-HSA-69563	p53-Dependent G1 DNA Damage Response	4	3.55E–03	0.0427
R-HSA-5658442	Regulation of RAS by GAPs	4	3.78E–03	0.0441
R-HSA-2467813	Separation of Sister Chromatids	7	3.83E–03	0.0441
R-HSA-1234176	Oxygen-dependent proline hydroxylation of Hypoxia-inducible Factor Alpha	4	4.01E–03	0.0441
R-HSA-5358346	Hedgehog ligand biogenesis	4	4.01E–03	0.0441
R-HSA-156902	Peptide chain elongation	5	4.24E–03	0.0451
R-HSA-174184	Cdc20:Phospho-APC/C mediated degradation of Cyclin A	4	4.26E–03	0.0451
R-HSA-68949	Orc1 removal from chromatin	4	4.26E–03	0.0451
R-HSA-68882	Mitotic Anaphase	8	4.34E–03	0.0451
R-HSA-2555396	Mitotic Metaphase and Anaphase	8	4.45E–03	0.0451
R-HSA-174178	APC/C:Cdh1 mediated degradation of Cdc20 and other APC/C:Cdh1 targeted proteins in late mitosis/early G1	4	4.51E–03	0.0451
R-HSA-179419	APC:Cdc20 mediated degradation of cell cycle proteins prior to satisfation of the cell cycle checkpoint	4	4.51E–03	0.0451
R-HSA-8948751	Regulation of PTEN stability and activity	4	4.51E–03	0.0451
R-HSA-202424	Downstream TCR signaling	5	4.62E–03	0.0454
R-HSA-69017	CDK-mediated phosphorylation and removal of Cdc6	4	4.77E–03	0.0454
R-HSA-975956	Nonsense Mediated Decay (NMD) independent of the Exon Junction Complex (EJC)	5	5.02E–03	0.0454
R-HSA-176409	APC/C:Cdc20 mediated degradation of mitotic proteins	4	5.05E–03	0.0454
R-HSA-5689880	Ub-specific processing proteases	7	5.09E–03	0.0458
R-HSA-156842	Eukaryotic Translation Elongation	5	5.22E–03	0.0470
R-HSA-75893	TNF signaling	4	5.33E–03	0.0480
R-HSA-176814	Activation of APC/C and APC/C:Cdc20 mediated degradation of mitotic proteins	4	5.33E–03	0.0480
R-HSA-69615	G1/S DNA Damage Checkpoints	4	5.33E–03	0.0480
